# Conjugated Polymer/Recombinant *Escherichia coli* Biohybrid Systems for Photobiocatalytic Hydrogen Production

**DOI:** 10.1021/acsnano.3c10668

**Published:** 2024-05-13

**Authors:** Ying Yang, Martijn A. Zwijnenburg, Adrian M. Gardner, Sylwia Adamczyk, Jing Yang, Yaqi Sun, Qiuyao Jiang, Alexander J. Cowan, Reiner Sebastian Sprick, Lu-Ning Liu, Andrew I. Cooper

**Affiliations:** † Materials Innovation Factory and Department of Chemistry, University of Liverpool, Liverpool L7 3NY, United Kingdom; ‡ Institute of Systems, Molecular and Integrative Biology, University of Liverpool, Liverpool L69 7ZB, United Kingdom; § Department of Chemistry, University College London, London WC1H 0AJ, United Kingdom; ∥ Stephenson Institute for Renewable Energy and the Department of Chemistry, University of Liverpool, Liverpool L69 7ZD, United Kingdom; ⊥ Early Career Laser Laboratory, University of Liverpool, Liverpool L69 3BX, United Kingdom; # Macromolecular Chemistry Group and Institute for Polymer Technology, Bergische Universität Wuppertal, Gauss-Straße 20, D-42097 Wuppertal, Germany; 7 Department of Pure and Applied Chemistry, University of Strathclyde, Glasgow G1 1XL, United Kingdom; 8 MOE Key Laboratory of Evolution and Marine Biodiversity, Frontiers Science Center for Deep Ocean Multispheres and Earth System & College of Marine Life Sciences, Ocean University of China, Qingdao 266003, China

**Keywords:** conjugated polymers, *Escherichia coli*, water splitting, biohybrid systems, biocatalysis

## Abstract

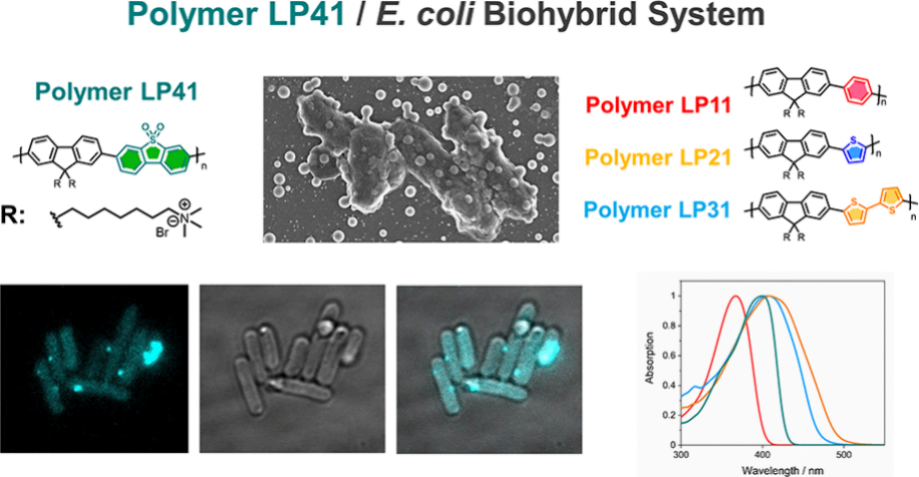

Biohybrid photocatalysts are composite materials that combine the efficient light-absorbing properties of synthetic materials with the highly evolved metabolic pathways and self-repair mechanisms of biological systems. Here, we show the potential of conjugated polymers as photosensitizers in biohybrid systems by combining a series of polymer nanoparticles with engineered *Escherichia coli* cells. Under simulated solar light irradiation, the biohybrid system consisting of fluorene/dibenzo [*b,d*]thiophene sulfone copolymer (LP41) and recombinant *E. coli* (i.e., a LP41/HydA BL21 biohybrid) shows a sacrificial hydrogen evolution rate of 3.442 mmol g^–1^ h^–1^ (normalized to polymer amount). It is over 30 times higher than the polymer photocatalyst alone (0.105 mmol g^–1^ h^–1^), while no detectable hydrogen was generated from the *E. coli* cells alone, demonstrating the strong synergy between the polymer nanoparticles and bacterial cells. The differences in the physical interactions between synthetic materials and microorganisms, as well as redox energy level alignment, elucidate the trends in photochemical activity. Our results suggest that organic semiconductors may offer advantages, such as solution processability, low toxicity, and more tunable surface interactions with the biological components over inorganic materials.

Generating clean and storable forms of energy from sunlight is a key challenge in the face of rising global energy demand and CO_2_-induced global warming. Photosynthetic organisms have evolved specialized photosynthetic machinery, comprising pigment–protein complexes and molecules, to capture sunlight and convert it into storable chemical energy, for example, sugars.^1−4^ However, natural photosynthesis is relatively inefficient, with a maximum photosynthetic energy conversion efficiency of approximately 4.5% calculated by Thorndike.^5^ As a consequence, extensive research has been dedicated to developing artificial photosynthetic systems, such as photovoltaic cells coupled to electrolyzers,^6^ photoelectrochemical cells,^7^ and photocatalysts.^8,9^ These artificial photosynthetic systems mimic biological systems by utilizing solar energy to power thermodynamically uphill reactions to generate storable fuels, such as hydrogen or formic acid.^10^

Artificial photosynthetic systems surpass some of the limitations of natural photosynthetic systems and can capture sunlight and drive fuel production more efficiently, mostly due to the development of highly efficient light-absorbing materials. On the other hand, biological organisms can facilitate electron transfer reactions and maintain sustainable repair and physiological regulation through active, multicomplex macromolecules.^11^

As such, there has been significant interest in integrating synthetic and biological systems to harness the strengths of both. This strategy, often known as semiartificial photosynthesis or biological–chemical hybrid photosynthesis, seeks to leverage the light-absorbing abilities of artificial photosynthetic systems with the dynamic regulation of metabolic pathways and self-regenerative abilities of biological systems.^12,13^ This field was initiated in the early 1980s when scientists combined TiO_2_ with *Clostridium butyricum*(14) and Bi_2_O_3_ with *Rhodopseudomonas capsulatus*, *Rhodospirilum rubrum*, and *E. coli*(15) to improve hydrogen production performance. Biohybrid systems have been studied more extensively since the beginning of this century. A range of inorganic semiconductors and complexes,^16,17^ such as CdS,^18−21^ AgInS_2_/In_2_S_3_,^22^ CdSexS_1–*x*_,^23^ Cu_2_O/reduced graphene oxide,^24^ and TiO_2_,^25−27^ have been coupled with *Shewanella oneidensis* and *E. coli* for hydrogen production. All these efforts provide necessary groundwork, although more fundamental research is required to establish their viability, not solely in the context of practical application.^28^

By contrast, organic semiconductors, such as conjugated polymers, are much less explored in biohybrid systems, despite their advantages such as the synthetically tunable optoelectronic properties and surface properties that are derived from a wide range of accessible monomers.^29^ Furthermore, diverse approaches have been explored to improve (photo)catalytic performances by saturating catalyst anchoring sites,^30^ enhancing surface hydrophobicity,^31^ and encapsulating catalysts.^32^ Combining conjugated polymers with biological systems also has the potential to overcome polymer photocatalysts’ reliance on co-catalysts, such as palladium and platinum, to improve photocatalytic proton reduction performance by providing alternative pathways using hydrogenases. Recently, organic photosensitizer eosin Y has been assembled with *E. coli* expressing [FeFe]-hydrogenase with^33,34^ and without^35^ the presence of redox mediators for hydrogen production. The intracellular location of the photosensitizer in this type of biohybrid system might offer efficient electron transfer to [FeFe]-hydrogenase, although screening of the photosensitizers is still needed for further improvement of the system. Furthermore, poly(fluorene-*co*-phenylene)^36,37^ and PFODTBT polymer dots^38^ have been reported as photosensitizers in microorganism-based biohybrid systems for CO_2_ reduction and nitrogen fixation, which allowed the development of polymer/bacteria biohybrid systems for the production of valuable chemicals.

Here, we assemble nanoparticles of conjugated linear polymers with *E. coli* cells mainly based on electrostatic interactions to develop a photobiocatalytic system for hydrogen production (Figure 1). The use of synthetic biology allowed us to access genetically engineered *E. coli* that can express [FeFe]-hydrogenase in addition to their endogenous [NiFe]-hydrogenase,^39^ while the use of conjugated polymers enabled us to tune the properties of the biohybrid via synthesis. This strategy significantly increases the proton reduction activity of the biohybrid system. The resulting biohybrid materials exhibited functional synergy between the two components and enhanced biohydrogen production by only using simulated solar light as the energy input.

**Figure 1:**
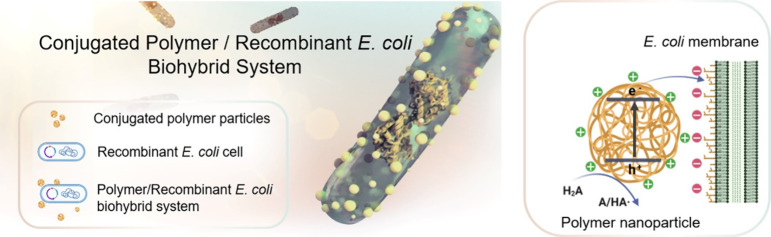
Strategy for the assembly of the conjugated polymer particle/recombinant *E. coli* biohybrid system mainly based on electrostatic interactions with one-hole (HA·/H_2_A) oxidation of ascorbic acid for hydrogen formation (3D rendering of an *E. coli* cell with nanoparticles on the surface copyright Dr. Thomas Fellowes; illustration of nanoparticle, *E. coli* cells, and biohybrid system created with BioRender.com with a publication license).

## Results/Discussion

Our approach to generating the polymer/*E. coli* biohybrid system involved the following steps: (i) constructing conjugated polymers using a range of building blocks to enable the absorption of visible light and the creation of charge carriers; (ii) modifying the polymers to establish strong electrostatic interactions with the bacterial cell membrane; (iii) preparing nanosized conjugated polymer particles with increased surface area to maximize polymer/cell interactions; (iv) integrating these nanoscale polymer particles with genetically engineered *E. coli* that express [FeFe]-hydrogenase in addition to their native [NiFe]-hydrogenase; and (v) using these biohybrid systems as photocatalysts for sacrificial hydrogen evolution.

### Assembly of the Conjugated Polymer/*E. coli* Biohybrid Systems

**Figure 2:**
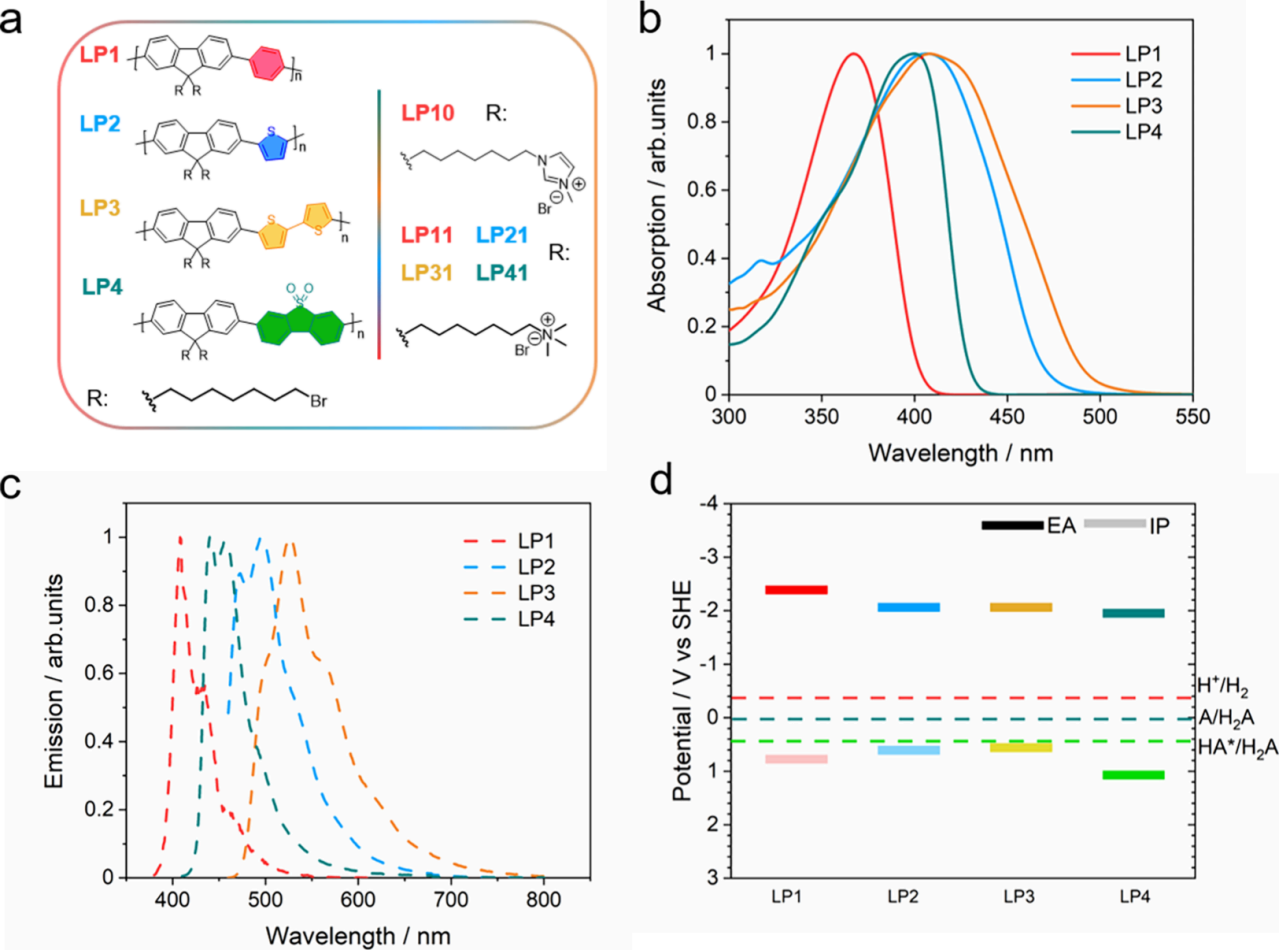
(a) Chemical structures of conjugated polymers LP1, LP2, LP3, and LP4 with imidazolium (LP10) and trimethylammonium (LP11, LP21, LP31, and LP41) functionalization. (b) Normalized UV–visible absorption spectra of polymers LP1, LP2, LP3, and LP4 dissolved in chloroform. (c) Normalized photoluminescence emission spectra of polymers LP1, LP2, LP3, and LP4 dissolved in chloroform. λ_exc_ = 370 nm for LP1, λ_exc_ = 400 nm for the rest of the polymers. (d) Predicted charge carrier potentials (IP, EA) of the polymers predicted through density functional theory (DFT) for oligomer models in water. Dashed colored lines indicate the potentials for different solution reactions: red, proton reduction; cyan and green, two-hole (A/H_2_A) and one-hole (HA·/H_2_A) oxidation of ascorbic acid. All solution potentials shown are for pH 6.5, which was the experimentally determined pH of a 10 mM tris(hydroxymethyl)aminomethane chloride buffer (pH 7, Tris-HCl) supplemented with 1 mM ascorbic acid.

Building on our experience with conjugated polymer photocatalysts for sacrificial hydrogen production from water in conjunction with palladium,^40,41^ we synthesized a series of copolymers with potential as visible light photocatalysts. Using Suzuki–Miyaura polycondensation reaction, we synthesized copolymers of bis(8-bromo-*n*-octyl)-fluorene (Figure 2a) with phenylene (LP1), thiophene (LP2), 2,2′-bithiophene (LP3), and dibenzo[*b,d*]thiophene sulfone (LP4) and purified them via Soxhlet extraction with methanol, acetone, and ethyl acetate. The polymers were then modified through polymer-analogous reactions on the alkyl-bromo functional groups to provide an imidazolium-substituted polymer (LP10)^42^ and trimethylammonium-substituted polymers (LP11, LP21, LP31, and LP41).^43^ The functionalization of the polymers with positively charged side groups was specifically designed to enable electrostatic interactions with the negatively charged outer cell membrane of *E. coli*.^44^

The polymers were characterized via ^1^H NMR, microanalysis, thermogravimetric analysis, gel permeation chromatography (GPC), and UV–visible (UV–vis) spectroscopy. GPC showed that the polymers had number-weighted molecular weights (*M*_n_) ranging from 13,900 to 125,900 g mol^–1^ (Table S1). All polymers exhibited visible light absorption (>400 nm), with the incorporation of thiophene units (LP2 and LP3) or dibenzo[*b,d*]thiophene sulfone (LP4) in place of phenylene (LP1), leading to significant red shifting of the absorption onset (Figures 2b, S3a). This redshift corresponds to a reduction in the optical gap (Table S2), and the broader peak of LP3 could be a result of the polymer chains having a greater degree of variation in both chain and conjugation lengths.^45^ All polymers are emissive ranging from 370 to 800 nm with Stokes shifts from 30 to 100 nm (Figures 2c, S3b).

The ionization potentials (IPs) of thin films of LP1–LP4 were measured using photoelectron spectroscopy in air (PESA; Table S4). It was not possible to perform these measurements with the imidazolium-substituted and trimethylammonium-substituted polymers due to their limited solubility that resulted in poor film formation. However, as the aromatic core of the materials is unchanged, no major differences in the ionization potentials would be expected. Density functional theory (DFT) was utilized to predict IP values using the B3LYP^46−73^ density functional for oligomer models of the polymers embedded in a dielectric continuum typical of organic solids (εr 2.0) (Figure 2d; Table S5). The results agreed with their PESA counterparts, in line with previous work.^41,49^ The DFT calculations and PESA measurements show that LP2 and LP3 have similar IP values, that LP1 has a deeper, more positive IP value, and that LP4 has the deepest, most positive IP value. Similar DFT calculations were made for oligomer models in water (εr 80.1), modeling a regime that is more difficult to probe experimentally (taken in the case of LP1 and LP4 from previous work),^50^ and these calculations showed the same trend.

**Figure 3:**
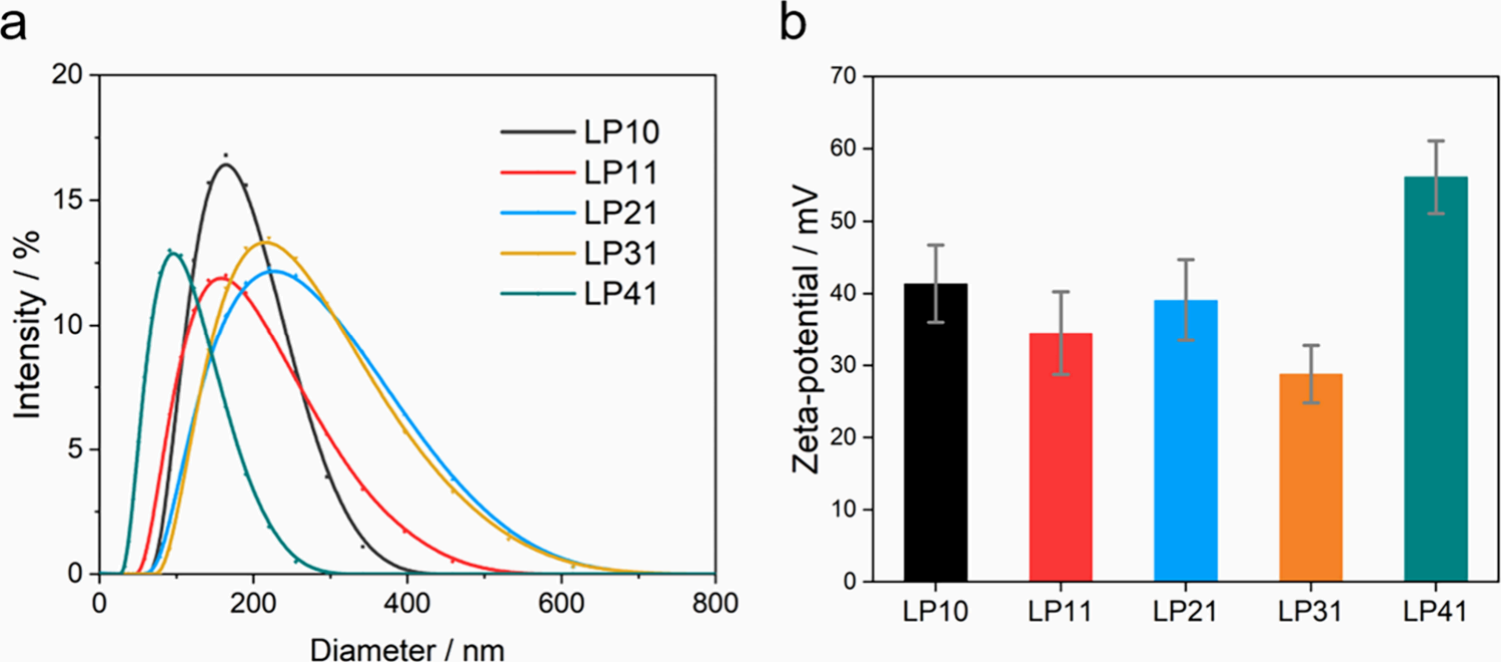
(a) Size distribution and (b) zeta-potential values (*n* = 3) of nanoparticle solutions of 50 mg L^–1^ polymer LP10, LP11, LP21, LP31, and LP41 by dynamic light scattering measurements.

**Figure 4:**
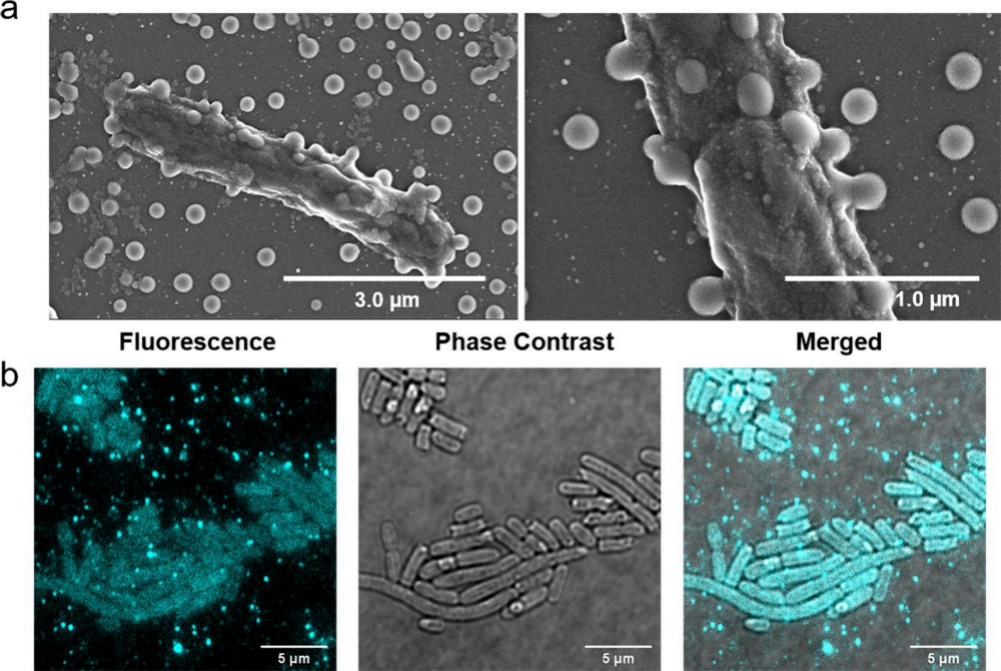
(a) SEM images of *E. coli* (200 μL in 10 mM Tris-HCl, optical density at 600 nm, OD_600_ ≈ 2.0) incubated with LP41 nanoparticles (2.0 mL, 10 mg L^–1^). (b) Confocal microscopy images of *E. coli* (100 μL in 10 mM Tris-HCl, OD_600_ ≈ 2.0) incubated with LP41 nanoparticles (1.0 mL, 5 mg L^–1^) for 5 min (λ_exc_ = 488 nm). 1 OD_600_ = 5 × 10^8^ cells mL^–1^ for *E. coli*.

The polymers were then processed into nanoparticles via a reprecipitation method.^51^ Size effects are important in photocatalysis because excitons do not typically propagate beyond 100 nm in conjugated organic materials.^52^ Larger particles therefore may have limited activities because excitons generated in the particle interior relax to the ground state before reaching the particle–solution interface. We also aimed to assemble polymer particles on bacteria, thus requiring that the polymer particles to be significantly smaller than the bacterial cell itself. Dynamic light scattering measurements showed that the polymer nanoparticles had diameters in the range of 95–258 nm with relatively broad size distributions, which is typical for conjugated polymer particles (Figure 3a, Figure S4, Table S3). LP41 was found to have the smallest particle size (95 nm) with a relatively spherical shape with a low degree of aggregation (Figure 4a). Although the particle preparation process was under the same conditions (polymer concentration and mixing ratio between tetrahydrofuran (THF) and water), the polymer chain conformation in THF and their interaction with THF might be different due to their different molecular weights (Table S1) and backbone structures. These might explain a variable morphology with varying degrees of aggregation in the solid state in scanning electron microscopy (SEM) images (Figure S5).^53^ Zeta-potentials were determined to be between +29 and +56 mV for the quaternary ammonium-functionalized polymer nanoparticles (Figure 3b), with LP41 having the highest zeta-potential value (56.1 ± 5.0 mV) among the materials studied here.

These polymer nanoparticles were then used to fabricate polymer/microorganism biohybrid systems. As a facultative anaerobe, wild-type (WT) *E. coli* BL21(DE3) can anaerobically synthesize endogenous [NiFe]-hydrogenase^54^ with the potential to produce hydrogen. To enhance hydrogen production, we generated an engineered *E. coli* strain by overexpressing the genes encoding [FeFe]-hydrogenase (HydA) and its maturases. This resulted in a highly active *E. coli* strain (HydA BL21) for hydrogen production, with 10–100 times greater activity for proton reduction compared to the native [NiFe]-hydrogenase.^55^

This *E. coli* strain was grown to express [FeFe]-hydrogenases (see Supporting Information for details),^39^ prior to the construction of the polymer/bacteria biohybrids (Figures S6 and S10). We found that the physiochemical properties of the materials such as polymer particle size, surface charge, and surface hydrophobicity/hydrophilicity are critical factors in the assembly process.^56^ Based on these observations, we assembled the recombinant *E. coli* (HydA BL21) with the polymer nanoparticles in a 10 mM tris(hydroxymethyl)aminomethane chloride buffer (pH 7, Tris-HCl) with 1 mM ascorbic acid after nitrogen purging. SEM and confocal images revealed that the polymer nanoparticles localized on the surface of the *E. coli* cells, while some free polymer nanoparticles were also present (Figure 4a and b).

### Photobiocatalytic Hydrogen Production Performance

**Figure 5:**
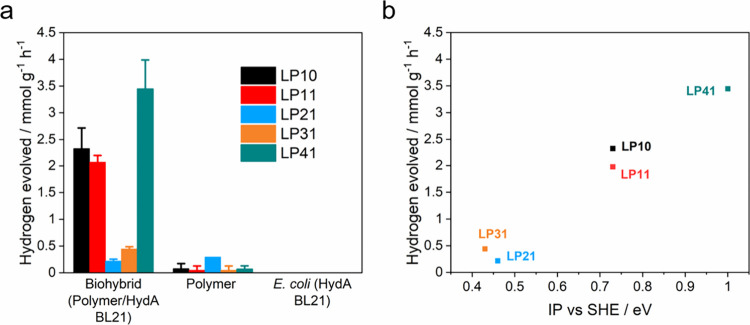
(a) Hydrogen production performance of biohybrid systems (4.3 mL 50 mg L^–1^ polymer nanoparticle solution, 200 μL of *E. coli* with OD_600_ ≈ 1.0) compared to the polymer nanoparticle and *E. coli* HydA BL21 control groups after 3 h of irradiation. (b) Correlation between the biohybrids’ hydrogen evolution activity and the polymer IP values. Plots and error bars represent the averages and standard deviations of at least two assays. All values were normalized to the polymer amount, and all measurements were conducted in 10 mM Tris-HCl buffer under irradiation of an AM 1.5G solar simulator.

The performance of the polymer/*E. coli* biohybrid systems for hydrogen production was evaluated in 10 mM Tris-HCl buffer supplemented with 1 mM ascorbic acid as the sacrificial agent under irradiation of an AM 1.5G solar simulator for 3 h. Biohybrid reactions in Figure 5a consisted of 4.3 mL of a 50 mg L^–1^ polymer nanoparticle solution and 200 μL of *E. coli* (optical density at 600 nm, OD_600_ ≈ 1.0, 1 OD_600_ = 5 × 10^8^ cells mL^–1^ for *E. coli*). The results showed that *E. coli* (HydA BL21) alone was found to be inactive and did not produce any detectable hydrogen, while the polymer nanoparticles alone produced only a small amount of hydrogen ranging from 4 nmol h^–1^ for LP41 to 13 nmol h^–1^ for LP21 (Figure 5a). The limited activity of these polymers on their own was attributed to the co-catalytic activity of residual palladium (Table S2) in the materials that originates from the Suzuki–Miyaura polycondensation reaction. When the polymer nanoparticles were coupled with the engineered *E. coli* cells, the amount of hydrogen produced significantly increased. The LP41 biohybrid system produced 148 nmol h^–1^, which was 31 times more than the polymer nanoparticles alone. The LP10 and LP11 biohybrid systems also showed increased hydrogen evolution, producing 100 and 89 nmol h^–1^, respectively. By contrast, the LP21 hybrid system showed a net reduction in hydrogen production (2 nmol h^–1^). The LP31 hybrid system showed only a small increase in hydrogen production (19 nmol h^–1^).

Consistent with our previous findings on conjugated polymer photocatalysts, there is little correlation between the observed hydrogen evolution rates and the polymers’ predicted electron affinity (EA) values, assuming that the potentials of the LP11–LP41 photocatalysts are similar to those of their LP1–LP4 counterparts. By contrast, there is a close correlation with the predicted IP values (Figure 5b). This is because the oxidation of the ascorbic acid electron donor is required for hydrogen evolution. All polymers are predicted to have a sufficiently negative EA, and hence a significant driving force for proton reduction, while the predicted IPs of polymers LP2 and LP3 are barely positive enough to drive ascorbic acid oxidation. There is also an apparent correlation with the polymer particles’ zeta-potential, most likely because the zeta-potential of the polymer particles varies in a similar fashion to their IP values. There is no clear correlation between the residual Pd content in each polymer (as indicated in Table S2) and the photocatalytic performance in both the polymer group and the biohybrid systems. For conjugated polymers, their photocatalytic hydrogen evolution activity dependence on residual Pd content is subject to specific polymer structures, because different quenching mechanisms would be involved for different backbones at different time scales.^57−59^ As such, we hypothesize that a similar scenario may apply to polymer biohybrid systems. However, in these systems, the increased hydrogen activity is likely attributed more to the hydrogenase in *E. coli* rather than the presence of Pd in the polymers.

**Figure 6:**
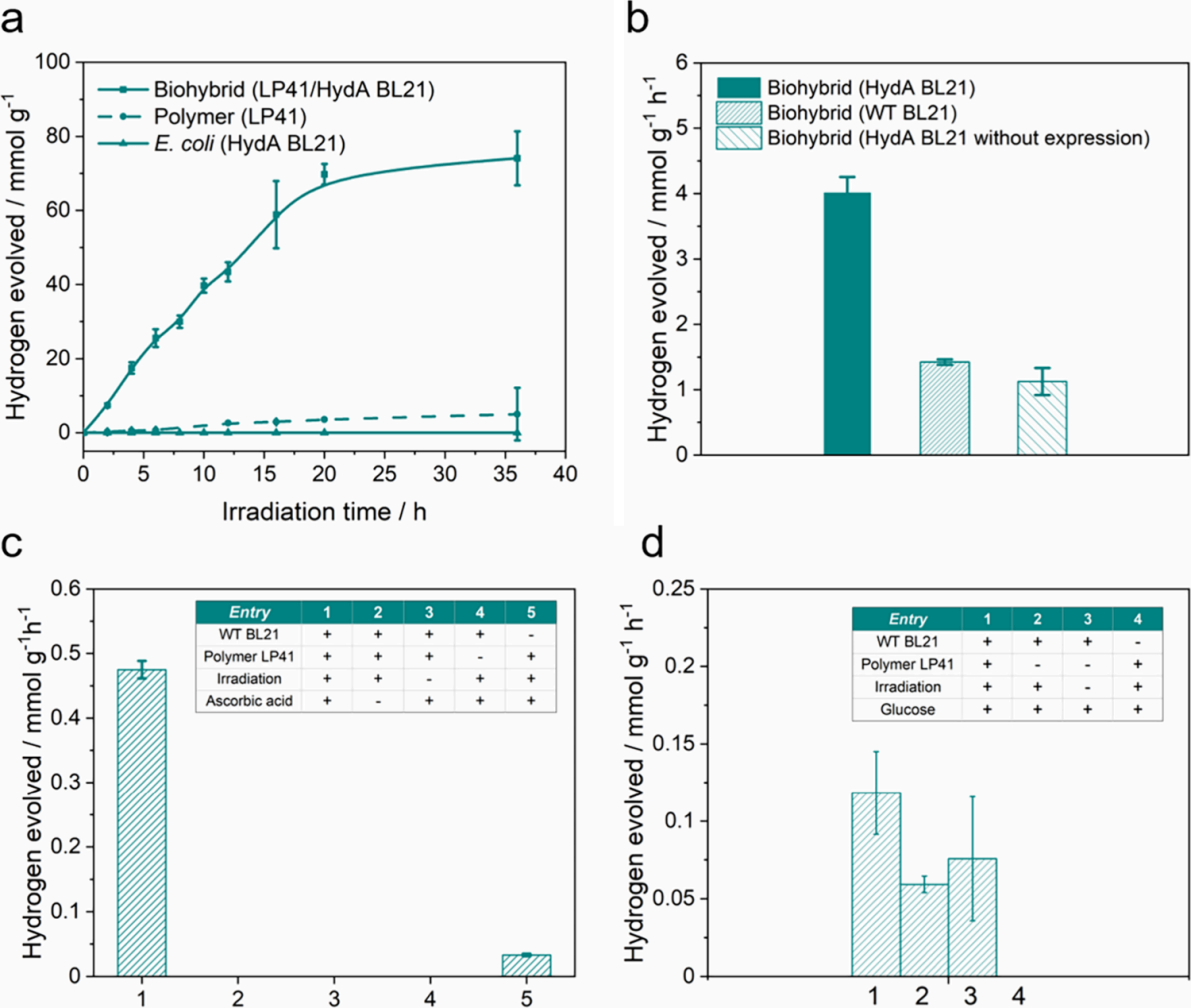
(a) Hydrogen evolution rate of the biohybrid (with 10 mg L^–1^ polymer LP41), the polymer nanoparticle (10 mg L^–1^ LP41), and *E. coli* (HydA BL21). (b) Hydrogen production performance of the LP41 nanoparticle coupled with HydA BL21, wild-type (WT) BL21, and HydA BL21 without [FeFe]-hydrogenase expression after a 3 h irradiation. Evolved hydrogen was normalized to the same cell concentration (OD_600_ = 1.0) for different *E. coli* strains. (c) Hydrogen production performance of LP41 coupled with WT *E. coli* compared with the biohybrid system without ascorbic acid (entry 1) and other controls (entries 2–5) after a 3 h reaction/irradiation. (d) Hydrogen production performance of LP41 coupled with WT *E. coli* with glucose and in the absence of ascorbic acid (entry 1) compared with other controls (entries 2–4) after a 3 h reaction/irradiation. Plots and error bars represent the averages and standard deviations of at least two assays. All values were normalized to the polymer amount, and all measurements were conducted in 10 mM Tris-HCl buffer under irradiation of an AM 1.5G solar simulator.

The polymer mass normalized LP41/*E. coli* biohybrid system has a photobiocatalytic sacrificial hydrogen evolution rate of 3.442 mmol g^–1^ h^–1^. Over 36 h, this biohybrid system produced 3334 nmol of hydrogen (Figure 6a). Steady hydrogen production observed for 20 h did not exhibit any significant rate change. By contrast, *E. coli* (HydA BL21) alone produced no measurable quantity of hydrogen, while the conjugated polymer LP41 produced only 452 nmol. Although there are very few estimates of the generation time of bacteria in biohybrid systems under photocatalytic conditions, *E. coli* can divide every 20 min in the laboratory under aerobic, nutrient-rich conditions.^60^ This might indicate that the physical interactions between *E. coli* cells and polymer particles in the biohybrid suspension are dynamic. It took approximately 12–16 h for *E. coli* to transfer from the logarithmic phase to stationary in optimal laboratory conditions (nutrient-rich media, 37 °C, and agitation),^61^ but no significant increase in cell density was observed in the biohybrid samples before and after irradiation (with an OD_600_ value around 1.0). However, it is important to note that the presence of polymer nanoparticles and exposure to irradiation could individually affect the sample turbidity, so caution should be taken with measured OD_600_ values for the interpretation of the growth phase. Apart from the cell growth phase, another important parameter that needs to be considered is the hydrogenase activity of *E. coli* under photocatalytic conditions. It was reported that there was a slight decrease in hydrogenase activity after a 24 h anaerobic induction, and it could potentially be attributed to the increased rates of cell death accompanied by protein degradation, as anoxic growth appeared to cease at this time.^62^

We also explored the role of [FeFe]-hydrogenase in HydA BL21 in the biohybrid system with the LP41 polymer (Figure 6b). When HydA BL21 without [FeFe]-hydrogenase expression was used in the biohybrid, a reduced hydrogen evolution rate of 50 nmol h^–1^ was observed compared to the biohybrid with expressed [FeFe]- hydrogenase (181 nmol h^–1^). The hydrogen evolution rate was similar to the biohybrid system containing *E. coli* WT BL21(DE3) (64 nmol h^–1^) that only expresses relatively less effective [NiFe]-hydrogenase. We, therefore, infer that the 3-fold increase in hydrogen evolution activity can be attributed to the expressed [FeFe]-hydrogenases in the biohybrid system, excluding the possibility that other factors, such as aggregation of the conjugated polymers, might be responsible for the increase in activity for hydrogen production and suggesting a photocatalytic process occurring between the conjugated polymer and *E. coli* cells.

Furthermore, the biohybrid system did not produce hydrogen under irradiation in the absence of ascorbic acid (Figure 6c, entry 2) and in the dark (Figure 6c, entry 3) after 3 h. In addition, the *E. coli* WT BL21 did not produce hydrogen with the presence of 1 mM ascorbic acid under a 3 h irradiation (Figure 6c, entry 4) and with other concentrations (0, 0.5, and 2.0 mM, Figure S12), which excludes the possibility that the sacrificial electron donor such as cysteine induced metabolic change for increased end product generation.^28^ Taken together, these results support the formation of a biohybrid system in which both components—the conjugated polymer and the genetically engineered *E. coli* cells—take part in a photocatalytic process. Nevertheless, when glucose was introduced as an energy and carbon source into the reaction mixture (without ascorbic acid), the hydrogen production performances of the biohybrid, WT *E. coli* under irradiation, and WT *E. coli* in the dark were nearly indistinguishable, yielding approximately 10–15 nmol of hydrogen after 3 h (Figure 6d). Although the hydrogen-producing activity through the glucose fermentation pathway was limited,^63^ it is reasonable to assume that *E. coli* was actively involved in hydrogen production in the presence of polymer LP41, even when residual Pd was present, during irradiation.

External quantum efficiency (EQE) under monochromatic light was estimated to be 0.08% at 395 nm and 0.05% at 420 nm (Table S10). These values are relatively low, but it should be noted that nanoparticle dispersions are optically clear, meaning that a significant amount of the incoming light passes through the sample without being absorbed. Comparisons in the saturated regime of catalyst concentration and with the same path length could be particularly useful measures of activity.^64,65^

### Interactions between the Conjugated Polymer and *E. coli*

To further understand the mechanism, particularly the nature of interaction between LP41 nanoparticles and *E. coli* cells, fluorescence intensity was measured as a function of the concentration of *E. coli* and ascorbic acid (Figure S14). It was found that both *E. coli* and ascorbic acid can quench the fluorescence intensities of LP41 although in a different manner. When *E. coli* was used as the quencher, there is a linear relationship between *I*_0_/*I* (the inverse of normalized emission intensity at 470 nm) and the relative equivalence of quenchers (*C*_quencher_/*C*_quencher,0_) from Stern–Volmer analysis (Table S7, Figure S15). This suggests dynamic quenching originating from diffusive encounters between *E. coli* and polymer particles during the lifetime of the photoexcited states.^66^ No obvious linear relationship was observed for LP41/*E. coli* with AA and LP41 with AA.^63^

Time-correlated single photon counting (TCSPC) was applied to obtain the fluorescence lifetimes. The estimated weighted average fluorescence lifetime of the nanoparticle of LP41 polymer in 10 mM Tris-HCl was reduced from 2.04 ns to 0.92 ns when 100 μL of WT *E. coli* in 10 mM Tris-HCl was added (Table S8, Figure S16). The addition of 2 mM ascorbic acid to the LP41/*E. coli* biohybrids with 50 μL of WT *E. coli* reduced the fluorescence lifetime from 1.21 ns to 0.91 ns. Likewise, the fluorescence lifetime of the conjugated polymer LP41 alone was reduced from 2.04 ns to 0.96 ns when 2 mM ascorbic acid was added.

However, it is often difficult to know the specific quenching mechanism, as they are not mutually exclusive, and quenching may occur by a combination of different mechanisms. The turbidity of samples increased when more *E. coli* was added, which might also cause decreased fluorescence intensities. In addition, there was no equivalent decrease in fluorescence intensities and lifetimes, which might indicate that the decreased fluorescence intensities were not caused by collisional quenching (alone).

To gain further insight into the LP41/*E. coli* biohybrid system, we performed transient absorption (TA) spectroscopy on the LP41 polymer and the LP41/*E. coli* biohybrid system (Figure S17). After excitation at 400 nm, the LP41 spectrum at 1 ps was dominated by a broad photoinduced absorption centered at 750 nm and a negative band at 500 nm, which is in good agreement with the emission spectrum of the sample (Figure S14c), and was assigned to stimulated emission. The decay of the broad band at 750 nm correlated with a decrease in stimulated emission and the growth of a band at 575 nm. We assigned the photoinduced absorption at 750 nm to a singlet excitonic species based on agreement with structurally related species^58^ and the correlation to the recovery of the stimulated emission band. The band at 575 nm was assigned to a charge transfer state at early times (∼1 ps) and electron polarons following hole scavenging, as for other similar polymers.^58^ Charge transfer states and polaron states such as polaron pairs and electron polarons are likely to have similar transient UV/visible spectra, making it hard to discriminate them. The 575 nm band appeared to first form within the instrument response function (∼1 ps) and continued to grow within the first 100 ps, consistent with reported ultrafast kinetics of similar polymers.^58^ At longer time scales of 100 ps to 3 ns, the remaining population of the initially formed excitonic state (750 nm center) decayed, in line with the TCSPC data that showed a lifetime of ∼2 ns.

The addition of ascorbic acid (dashed lines in Figure S17a) resulted in no change of the nature of the photogenerated species on the picosecond time scale, with both the photoinduced absorptions at 750 and 575 nm assigned to the initially formed singlet exciton and charge transfer/polaron state. Notably, the relative ratios of the 750 and 575 nm bands change in the presence of the ascorbic acid < 1 ns, with the yield of the 750 nm band being greater. We note that the presence of ascorbic acid may not only introduce an electron donor but also modify the solvent/reaction environment with the potential for direct interactions between the ascorbic acid and LP41 that might give rise to changes in emission yields and lifetimes. TA spectra showed that on the picosecond time scale, ascorbic acid did not change the rate/yield of the charge transfer state/polaron pair species at 575 nm, but the photocatalysis data showed clearly that ascorbic acid is required for activity. We therefore conclude that ascorbic acid may play a role in quenching the charge transfer state to prevent recombination on the nanoscale and slower time scales and that electron transfer from LP41 occurs on the time scales that are slower than studied here.

TA spectra recorded for LP41/*E. coli* samples (Figure S17b) supported the conclusion that electron transfer to *E. coli* BL21(DE3) was slow. These showed that the photoinduced absorption at 575 nm is retained to 3 ns (the longest time scale that can be studied here) in the presence of *E. coli* BL21(DE3).

## Conclusions

In this study, nanoparticles of a series of conjugated polymers with different backbone structures were assembled with engineered *E. coli* cells that express [FeFe]-hydrogenase, and the formed biohybrid systems were shown to be active for sacrificial hydrogen production from ascorbic acid solutions. The polymer/*E. coli* biohybrid systems were significantly more active than either the polymer nanoparticles or the *E. coli* cells in isolation under the same conditions. The biohybrid of LP41 (a copolymer of fluorene and dibenzo[*b,d*]thiophene sulfone) and *E. coli* was the most active material studied, with an absolute hydrogen evolution rate of 148 nmol h^–1^. Compared with the other polymers in this study, LP41 had a favorable driving force for hole scavenger oxidation, an appropriate particle size, and a positive surface charge that resulted in the formation of a biohybrid system with higher activity.

However, we emphasize that the direct comparison of the hydrogen evolution performances between our biohybrid system and other previously reported biohybrids (Table S9) is not possible. This is because various light sources and optical setups are used and also because of the use of different reaction solutions, especially for those with glucose as an additional energy source.^19,22^ In those systems, photogenerated electrons from materials under irradiation are not the only electron source, as glucose can also act as an electron donor for fermentative hydrogen production.^19,63^ Furthermore, stability is the other important indicator in evaluating the biohybrid systems, which is mainly limited by the activity of the microorganisms involved. Using a flow system equipped with automation to separate materials from biohybrid suspension and replace with fresh cell culture at regular intervals might provide opportunities to address this challenge.

Our study also highlights the crucial role of the hydrogenase type expressed in *E. coli*. PL lifetime and TA studies suggest, although do not prove, charge transfer between the conjugated polymer and the bacterial cells under irradiation. Overall, this study adds to our fundamental understanding of this type of organic biohybrid system. While these systems are far from practical, not least because of the use of a sacrificial hole scavenger, the results do suggest that organic semiconductors are equally viable for biohybrid photocatalyst manufacture and that they may offer certain advantages over inorganic materials, such as low toxicity, engineerable surface properties, and solution processability.

## Methods/Experimental

### Monomer Synthesis

#### 2,7-Dibromo-9,9-bis(8-bromo-*n*-octyl)fluorene

The compound was synthesized following a previously reported procedure.^42^ NaOH (30 mL, aqueous solution 50 wt %) was added to a solution of 2,7-dibromo-9*H*-fluorene (5.0 g, 15 mmol), 1,8-dibromo-*n*-octane (8.5 mL, 46 mmol), and tetrabutylammonium bromide (TBAB, 0.5 g, 1.5 mmol) in toluene (60 mL). The mixture was then heated to 60 °C for 12 h. After cooling to room temperature, phases were separated, and the aqueous phase was extracted with chloroform. The combined organic phase was washed with deionized water, dried with magnesium sulfate, filtered, and concentrated *in vacuo* to give residues, which were then purified by silica gel column chromatography, ethyl acetate/hexane = 1:50 by volume ratio, to obtain a colorless solid (6.3 g, 59%). ^1^H NMR (400 MHz, CDCl_3_, δ): 7.52 (d, 2H, ^3^*J* = 8 Hz, Ar–H), 7.46 (dd, 2H, ^4^*J* = 2 Hz, ^3^*J* = 8 Hz, Ar–H), 7.43 (d, 2H, ^4^*J* = 2 Hz, Ar–H), 3.35 (t, 4H, ^3^*J* = 8 Hz; −CH_2_Br), 1.91 (m, 4H; −CH_2_−), 1.77 (m, 4H; −CH_2_−), 1.31 (m, 4H; −CH_2_−), 1.14–1.05 (br, 12H; −CH_2_−), 0.57 (br, 4H; −CH_2_−).

### General Procedure for the Synthesis of Polymers via Suzuki–Miyaura-Type Polycondensation

A flask was charged with the monomers, toluene, Starks’ catalyst, and an aqueous solution of Na_2_CO_3_. The mixture was degassed by bubbling with N_2_ for 30 min, before [Pd (PPh_3_)_4_] was added, and heated. The mixtures were evaporated to dryness and washed with water. The crude polymer was then further purified by Soxhlet extraction with methanol, acetone, and ethyl acetate. The high molecular weight fraction of the polymer was recovered by Soxhlet extraction with chloroform. The chloroform was removed and the polymer redissolved in a minimal amount of chloroform, precipitated into a large excess of methanol, filtered off, and dried under reduced pressure.

#### Synthesis of LP1

1,4-Benzenediboronic acid bis(pinacol) ester (264 mg, 0.80 mmol), 2,7-dibromo-9,9-bis(8-bromo-*n*-octyl)fluorene (567 mg, 0.80 mmol), toluene (30 mL), Na_2_CO_3_ (10 mL, 2 M), Starks’ catalyst (1 drop), and [Pd (PPh_3_)_4_] (18.6 mg) were used in this reaction. After 2 days at 110 °C the reaction was worked up as described above, giving the product as a yellow solid in 55% yield (0.457 g). Anal. Calcd for LP1 (C_35_H_44_Br_2_)_*n*_: C, 67.31; H, 7.10; Br, 25.59%. Found: C, 67.62; H, 6.72; Pd, 0.17%. ^1^H NMR (400 MHz, CDCl_3_, δ): 7.82 (m, 6H, Ar–H), 7.66–7.49 (m, 4H, Ar–H), 3.32 (t, 4H, ^3^*J* = 6 Hz, −CH_2_Br), 2.09 (br, 4H, −CH_2_−), 1.75 (m, 4H, −CH_2_−), 1.31–1.12 (m, 16H, −CH_2_−), 0.78 (br, 4H, −CH_2_−).

#### Synthesis of LP2

2,5-Thiophenediboronic acid bis(pinacol) ester (268.8 mg, 0.80 mmol), 2,7-dibromo-9,9-bis(8-bromo-*n*-octyl)fluorene (567 mg, 0.80 mmol), toluene (30 mL), Na_2_CO_3_ (10.0 mL, 2 M), Starks’ catalyst (1 drop), and [Pd (PPh_3_)_4_] (18.6 mg) were used in this reaction. After 2 days at 110 °C the reaction was worked up as described above, giving the product as an orange solid in 21% yield (0.175 g). Anal. Calcd for LP2 (C_33_H_42_Br_2_S)_*n*_: C, 62.86; H, 6.71; Br, 25.34; S, 5.08%. Found: C, 69.45; H, 6.79; S, 3.80, Pd, 0.44%. ^1^H NMR (400 MHz, CDCl_3_, δ): 7.72–7.56 (m, 6H, Ar–H), 7.48–7.40 (m, 2H, Ar–H), 3.32 (m, 4H, −CH_2_Br), 2.01 (br, 4H, −CH_2_−), 1.75 (m, 4H, −CH_2_−), 1.28–1.09 (m, 16H, −CH_2_−), 0.65 (br, 4H, −CH_2_−).

#### Synthesis of LP3

2,2′-Bithiophene-5,5′-diboronic acid bis(pinacol) ester (334.5 mg, 0.80 mmol), 2,7-dibromo-9,9-bis(8-bromo-*n*-octyl)fluorene (567 mg, 0.80 mmol), toluene (30 mL), Na_2_CO_3_ (10.0 mL, 2 M), Starks’ catalyst (1 drop), and [Pd (PPh_3_)_4_] (18.6 mg) were used in this reaction. After 2 days at 110 °C the reaction was worked up as described above, giving the product as a coral solid in 44% yield (0.397 g). Anal. Calcd for LP3 (C_37_H_44_Br_2_S_2_)_*n*_: C, 62.36; H, 6.22; Br, 22.42; S, 9.00%. Found: C, 64.89; H, 6.10; S, 8.13, Pd, 0.0093%. ^1^H NMR (400 MHz, CDCl_3_, δ): 7.70–7.46 (m, 6H, Ar–H), 7.34–7.20 (m, 4H, Ar–H), 3.31 (m, 4H, −CH_2_Br), 2.01 (br, 4H, −CH_2_−), 1.74 (m, 4H, −CH_2_−), 1.28–1.09 (m, 16H, −CH_2_−), 0.69 (br, 4H, −CH_2_−).

#### Synthesis of LP4

3,7-Bis(4,4,5,5-tetramethyl-1,3,2-dioxaborolan-2-yl)dibenzo[*b*,*d*]thiophene sulfone (374.5 mg, 0.80 mmol), 2,7-dibromo-9,9-bis(8-bromo-*n*-octyl)fluorene (567 mg, 0.80 mmol), toluene (30 mL), Na_2_CO_3_ (10.0 mL, 2 M), Starks’ catalyst (1 drop), and [Pd(PPh_3_)_4_] (18.6 mg) were used in this reaction. After 2 days at 110 °C the reaction was worked up as described above, giving the product as a light-yellow solid in 36% yield (0.122 g). Anal. Calcd for **LP4** (C_41_H_46_Br_2_O_2_S)_*n*_: C, 64.57; H, 6.08; Br, 20.95; O, 4.20; S, 4.20%. Found: C, 65.98; H, 5.74; S, 3.16, Pd, 0.027%. ^1^H NMR (400 MHz, CDCl_3_, δ): 7.70–7.46 (m, 6H, Ar–H), 7.34–7.20 (m, 6H, Ar–H), 3.33 (t, 4H, ^3^*J* = 8 Hz, −CH_2_Br), 2.13 (br, 4H, −CH_2_−), 1.76 (m, 4H, −CH_2_−), 1.35–1.05 (m, 16H, −CH_2_−), 0.71 (br, 4H, −CH_2_−).

### General Procedure for the Synthesis of Imidazolium-Substituted Polymers^42^

A 0.1 g amount of LP1 was dissolved in N_2_-purged toluene (40 mL), and 1-methylimidazole (2 g, 24 mmol) was added dropwise. After the reaction was stirred at 40 °C for 12 h, MeOH (100 mL) was added to dissolve precipitated polymer, and the reaction was stirred at 40 °C for 5 days. After the solution was concentrated in vacuo, the residue was poured into 100 mL of ethyl acetate and precipitates were filtered, washed with acetone several times, and dried in a vacuum oven overnight at room temperature to obtain imidazolium-substituted yellow solid LP10 in 74% yield (74 mg). Anal. Calcd for LP10 (C_43_H_50_N_4_)_*n*_: C, 82.12; H, 8.97; N, 8.91%. Found: C, 64.61; H, 7.09; N, 2.88; Pd, 0.16%. ^1^H NMR (400 MHz, DMSO, δ): 8.10–7.60 (m, 16H, Ar–H), 4.06 (br, 4H, −CH_2_−), 3.81 (br, 4H, −CH_2_−), 1.65 (s, 4H, −CH_2_−), 1.31–0.50 (m, 20H, −CH_2_−).

### General Procedure for the Synthesis of Trimethylammonium-Substituted Polymers^43^

A 0.1 g amount of LP1, LP2, LP3, and LP4 were dissolved in CHCl_3_ (20 mL), and then a solution of trimethylamine in ethanol (30 wt %, 10 mL) was added. The reaction mixture was stirred for 48 h at room temperature. The solvent was evaporated, and the product was dried under vacuum.

#### LP11:

yellow solid in 78% yield (78 mg). Anal. Calcd for LP11 (C_41_H_62_N_2_)_*n*_: C, 84.47; H, 10.72; N, 4.81%. Found: C, 63.76; H, 7.74; N, 2.88; Pd, 0.16%. ^1^H NMR (400 MHz, DMSO, δ): 8.20–7.40 (m, 10H, Ar–H), 3.36 (s, 4H, −CH_2_−), 3.22 (br, 4H, −CH_2_−), 3.01 (m, 18H, −CH_3_), 1.55 (s, 4H, −CH_2_−), 1.31–0.85 (m, 16H, −CH_2_−), 0.65 (br, 4H, −CH_2_−).

#### LP21:

orange solid in 45% yield (45 mg). Anal. Calcd for LP21 (C_39_H_60_N_2_S)_*n*_: C, 79.53; H, 10.27; N, 4.76; S, 5.44%. Found: C, 62.85; H, 7.73; N, 2.95; S, 3.37; Pd 0.43%. ^1^H NMR (400 MHz, DMSO, δ): 8.12–7.10 (m, 8H, Ar–H), 3.33 (s, 4H, −CH_2_−), 2.98 (s, 18H, −CH_2_−), 2.09 (s, 4H, −CH_2_), 1.62–0.30 (m, 24H, −CH_2_−).

#### LP31:

coral solid in 58% yield (58 mg). Anal. Calcd for LP31 (C_43_H_62_N_2_S_2_)_*n*_: C, 76.96; H, 9.31; N, 4.17; S, 9.55%. Found: C, 62.43; H, 7.34; N, 2.81; S, 6.91; Pd, 0.0093%. ^1^H NMR (400 MHz, DMSO, δ): 8.15–7.10 (m, 10H, Ar–H), 3.36 (s, 4H, −CH_2_−), 3.17 (br, 4H, −CH_2_−), 2.99 (m, 18H, −CH_3_), 1.54 (s, 4H, −CH_2_−), 1.38–0.37 (m, 20H, −CH_2_−).

#### LP41:

light-yellow solid, 71% yield (71 mg). Anal. Calcd for LP41 (C_47_H_64_N_2_O_2_S)_*n*_: C, 78.29; H, 8.95; N, 3.88; O, 4.44; S, 4.45%. Found: C, 60.67; H, 6.66; N, 2.64; S, 3.43%; Pd, 0.021%. ^1^H NMR (400 MHz, DMSO, δ): 8.65–7.80 (m, 12H, Ar–H), 3.35 (s, 4H, −CH_2_−), 3.16 (br, 4H, −CH_2_−), 2.98 (m, 18H, −CH_3_), 1.53 (s, 4H, −CH_2_−), 1.38–0.35 (m, 20H, −CH_2_−).

### Conjugated Polymer Nanoparticle Preparation

Conjugated polymers were self-assembled into polymer (nano)particles in water through reprecipitation methods.^51^ To achieve this, the conjugated polymer, LP10, LP11, LP21, LP31, and LP41 (10 mg), was dissolved by stirring in THF (12.5 mL). A 3 mL amount of the polymer/THF solution was added quickly to 12 mL of deionized water while sonicating the mixture under 30 °C for around 10 s. The THF was removed by partial evaporation at 65 °C for 5 h, followed by filtration through a 0.45 μm Nylon syringe filter.

### Expression of [FeFe]-Hydrogenase and Its Maturases in *E. coli* BL21

The expression of mature, functional [FeFe]-hydrogenases, as well as ferredoxin, was conducted following the procedure reported in our previous work.^39^*E. coli* BL21(DE3) cells containing the *hyd* vector were first grown aerobically in lysogeny broth (LB) medium containing 0.2 mM ferric ammonium citrate and 50 μg mL^–1^ spectinomycin at 37 °C until OD_600_ reached 0.7–0.8. Cells were then transferred to Falcon tubes sealed with rubber turnover closures and degassed with nitrogen for 15 min before the addition of 0.5 mM IPTG, 0.2 mM l-cysteine, and 2.5 mM sodium fumarate for anaerobic treatment. Cells were then grown at 25 °C for 16 h.

### Scanning Electron Microscope Characterization of *E. coli* Incubated with Polymer Nanoparticles

*E. coli* was harvested by centrifuging at 7197 rcf (relative centrifugal force) for 5 min and washed with 10 mM Tris-HCl buffer 3 times. After the supernatant was discarded, the cell pellet was resuspended in 10 mM Tris-HCl. Then 200 μL of cell suspension (final OD_600_ ≈ 1.0) was incubated with 2.0 mL of 10 mg L^–1^ polymer nanoparticle solution for 10 min at room temperature. To prepare a specimen for SEM characterization, 10 μL of the mixture was pipetted on top of a silica disc which was mounted on a metal stub by silver-containing glue. Then the dried specimen was coated with chromium for 15 s by Quorum Q150T ES. SEM measurements were performed on a Hitachi S4800 cold field emission scanning electron microscope (FE-SEM), and imaging was conducted at a working voltage of 3.0 kV and a working distance of 8 mm using a combination of upper and lower secondary electron detectors.

### Confocal Fluorescence Microscope Characterization of *E. coli* Incubated with Polymer Nanoparticles

*E. coli* cells were harvested by centrifuging at 7197*g* for 5 min and washed with 10 mM Tris-HCl buffer 3 times. After the supernatant was discarded, the cell pellet was resuspended in 10 mM Tris-HCl, pH 7. Then 100 μL of cell suspension (final OD_600_ ≈ 1.0) was incubated with 1.0 mL of 5 mg L^–1^ polymer nanoparticle solution for 10 min at room temperature. To immobilize the cells for imaging, 10 μL of the mixture was pipetted on top of Tris-HCl/agar, and a dried drop was cut out and placed against a coverslip.^69−71^ Live-cell confocal fluorescence imaging was performed on an LSM 780 microscope (Zeiss) with a 63× oil-immersion objective (numerical aperture: 1.46), and excitation at 488 nm was used in imaging. Images were processed with the FIJI image processing package.

### Theoretical Calculations

The IP and EA values of LP2 and LP3 were predicted using a previously developed approach^72,49^ based on DFT calculations on the neutral, cationic, and anionic versions of oligomer models of the polymers. These calculations used the B3LYP^46−73^ density functional, the DZP^74^ basis-set, and the COSMO^75^ implicit solvation model to describe the dielectric environment of the polymer (particles). All DFT calculations were performed using the Turbomole^76,77^ 7.5 code, and all calculations were performed on 8 phenyl equivalent long oligomeric models. Starting structures for the latter were obtained using a CREST/gfn2-xTB^78,79^ conformer search.

### Transient Absorption Measurements

The apparatus employed to obtain transient absorption and spectra of the suspensions of interest has been recently reported.^80^ Briefly, ∼1 W from an Ytterbium laser system (PHAROS Short-Pulse 10 W, PH1-SP-10W, Light Conversion) with an output wavelength of 1028 nm, a repetition rate of 10 kHz, and pulse duration of ∼170 fs is used to drive an optical parametric amplifier, OPA (ORPHEUS, Light Conversion) in tandem with a second-harmonic generation module (LYRA, Light Conversion) in order to generate radiation centered at 400 nm with a bandwidth (fwhm) of 3 nm. This 400 nm output was used as the pump source for subsequent TA measurements, which employed a commercial TA spectrometer (HARPIA, Light Conversion). The probe light was a visible white light supercontinuum generated by focusing <0.1 W of 1028 nm radiation onto a sapphire window. The pump and probe beams were focused to 1 mm and 600 μm spots at the sample. The pump laser beam was chopped, resulting in an effective pumping repetition rate of 5 kHz. The power of the chopped beam incident on the sample was 200 μW. The samples were suspensions of LP41 polymer nanoparticle (∼10 mg L^–1^ LP41 polymer concentration) in 10 mM Tris-HCl buffer with/without 25 μL of *E. coli* supplemented with/without 1 mM ascorbic acid, held within a quartz cuvette with a 10 mm path length. To maintain a stable suspension and to prevent sample degradation, the sample was continually stirred. The probe light was spectrally dispersed by a spectrograph (Kymera 193i, Andor), employing a grating of 150 lines/mm, blazed at 800 nm, and detected using an NMOS detector (S3901, Hamamatsu). Data were analyzed using Carpetview software (Light Conversion); all data were normalized to the global maximum Δ*A* to account for changes in UV/vis absorption spectra between different samples.

### Photobiocatalytic Hydrogen Production Measurements

For the photocatalytic hydrogen production measurements, a suspension of recombinant *E. coli* cells was prepared as follows: Cells were harvested from 20 mL of cell solution by centrifugation (10 min, 4000 rcf) after 16 h of induction. After the supernatant was carefully removed by syringes, the cell pellet was then resuspended in 1.0 mL of 10 mM Tris-HCl (pH 7) buffer after washing with 10 mL of 10 mM Tris-HCl buffer three times.

For high-throughput solar simulator measurements, 4.3 mL of 10 or 50 mg L^–1^ polymer nanoparticle solution, 0.5 mL of 100 mM Tris-HCl (pH 7) buffer, and 50 μL of 0.1 M ascorbic acid were added into headspace vials (Agilent, 10 mL, width 22.75 × height 46 mm) and purged with nitrogen in a Sweigher Chemspeed Technologies for 6 h. After 200 μL of collected cell suspension (final OD_600_ ≈ 1.0) was injected into vials, all sample vials were irradiated under the solar simulator (AM 1.5G, Class AAA, IEC/JIS/ASTM, 1440 W xenon, 12 × 12 in., model 94123A) agitated on a rocker/roller device. Gaseous products were analyzed on a Shimadzu GC-2010 equipped with a Shimadzu HS-20, injecting the sample from the headspace sampler via a transfer line (temperature 150 °C) onto a Rt-Msieve 5 Å column with He as the carrier gas at a flow rate of 30 mL min^–1^. Hydrogen was detected with a barrier discharge ionization detector referencing against standard gases with known concentrations of hydrogen.

### External Quantum Efficiency Measurements

A 6.8 mL amount of 10 mg/L LP41, 0.8 mL of 100 mM Tris-HCl (pH 7), and 80 μL of 0.1 M ascorbic acid were added to a quartz cuvette (8 mL not including headspace volume, face area: 4 cm × 2 cm) fitted with a magnetic stirrer bar. The cuvette was then sealed with an airtight rubber septum and degassed with nitrogen for 20 min after 300 μL of HydA *E. coli* BL21 pellet was injected.

The cuvette was then illuminated in turn using LEDs of specific wavelengths (395, 420 nm). The power outputs of LED at specific wavelengths were determined using a Thorlabs optical energy meter. Total hydrogen evolution was determined using GC after illumination after a 90 min period.

## Supporting Information Available

Experimental details, GPC, PXRD, TGA, UV–vis spectra, PL spectra, SLS data, SEM images, confocal images, PESA data, (TD-)DFT data, photocatalysis data, and TRSPC data (PDF)
DFT structures (ZIP)

## ABBREVIATIONS

*E. coli*: *Escherichia coli*
GPC: gel permeation chromatography
PESA: photoelectron spectroscopy in air
DFT: density functional theory
IP: ionization potential
EA: electron affinity
OD_600_: optical density at 600 nm
Tris-HCl: tris(hydroxymethyl)aminomethane chloride
WT: wild-type
TCSPC: time-correlated single-photon counting
TA: transient absorption
