# Supplementary material for: Conjugated Polymer/Recombinant Escherichia coli Biohybrid Systems for Photobiocatalytic Hydrogen Production
Source: ACS Nano. 2024 May 13;18(21):13484–95. doi: 10.1021/acsnano.3c10668 (PMC11140839; doi:10.1021/acsnano.3c10668)
Supplement: Supplementary file 1 — nn3c10668_si_001.pdf [file nn3c10668_si_001.pdf]

Conjugated Polymer/Recombinant *Escherichia coli* Biohybrid Systems for Photobiocatalytic Hydrogen Production

Ying Yang,[1,2] Martijn A. Zwijnenburg,[3] Adrian M. Gardner,[4,5] Sylwia Adamczyk,[6] Jing Yang,[1,2] Yaqi Sun,[2] Qiuyao Jiang,[2] Alexander J. Cowan,[4,5] Reiner Sebastian Sprick,[7] Lu-Ning Liu,[2,8*] Andrew I. Cooper[1*]*

[1] Materials Innovation Factory and Department of Chemistry, University of Liverpool, Liverpool L7 3NY, United Kingdom.

[2] Institute of Systems, Molecular and Integrative Biology, University of Liverpool, Liverpool L69 7ZB, United Kingdom.

[3] Department of Chemistry, University College London, London, WC1H 0AJ United Kingdom.

[4] Stephenson Institute for Renewable Energy and the Department of Chemistry, University of Liverpool, Liverpool L69 7ZD, United Kingdom.

[5] Early Career Laser Laboratory, University of Liverpool, Liverpool L69 3BX, United Kingdom.

[6] Macromolecular Chemistry Group and Institute for Polymer Technology, Bergische Universität Wuppertal, Gauss-Str. 20, D-42097 Wuppertal, Germany.

[7] Department of Pure and Applied Chemistry, University of Strathclyde, Glasgow G1 1XL, United Kingdom.

[8] MOE Key Laboratory of Evolution and Marine Biodiversity, Frontiers Science Center for Deep Ocean Multispheres and Earth System & College of Marine Life Sciences, Ocean University of China, Qingdao 266003, China.

Table of Content

Table of Content 2
Experimental Procedures 3
Materials and Instruments...3
Results and Discussion 3
Gel Permeation Chromatography 3
Photophysical Properties and Residual Pd Content 4
Powder X-Ray Diffraction...5
Thermogravimetric Analysis 5
UV-Vis and Photoluminescence Spectra 6
Size Distribution and Zeta-potential 7
Scanning Electron Microscopy..8
Confocal Fluorescence Microscopy...10
Photoelectron Spectroscopy in Air (PESA) 11
(TD-)DFT Calculations 12
Photobiocatalytic Hydrogen Production..13
Photoluminescence Spectra 16
Stern-Volmer Analysis 17
Time Correlated Single Photon Counting..19
Transient Absorption Measurements 21
Previous Literature Reports on Material-Microorganism Biohybrids for Hydrogen Production............23
External Quantum Efficiency (EQE) Measurements 24
References 25

Experimental Procedures

Materials and Instruments

Reagents and solvents were purchased from Manchester Organics, Sigma-Aldrich, Fluorochem, Ark Pharm, Apollo, Combi-Blocks, TCI Europe, Carbosynth and used as received without further purification.

^{1}H NMR spectra were recorded using a Bruker Avance 400 NMR spectrometer. CHN Analysis was performed on a Thermo EA1112 Flash CHNS-O Analyzer using standard microanalytical procedures. Single detection gel permeation chromatography (GPC) was calibrated against polystyrene standards (Agilent EasiCal PS-2 standards) and performed using an Agilent 1260 Infinity II GPC/SEC system (Agilent, UK), two PLgel 5 μm MIXED-D columns and a PL gel 5 μm guard column, with samples detected by refractive index (RI). Powder X-ray diffraction (PXRD) measurements were performed on a PANalytical X'Pert PRO MPD, with a Cu X-ray source, used in high throughput transmission mode with Kα focusing mirror and PIXCEL 1D detector. Thermogravimetric analysis was performed on an EXSTAR6000 by heating samples at 10 °C min^{-1} under nitrogen in open platinum pans from 25 to 600 °C. UV-Visible absorption spectra of polymer nanoparticle dispersions and polymer solutions in chloroform were recorded on a Shimadzu UV-2550 UV-Vis spectrometer. Photoluminescence spectra of the polymer nanoparticle solution were measured with a Shimadzu RF-5301PC fluorescence spectrometer at room temperature. Dynamic light scattering and zeta-potential measurements were performed on a Malvern Zetasizer Nano Particle Sizer at 25 °C. Inductively coupled plasma optical emission spectrometry analysis was performed on an ICP-OES Agilent 5110 after a microwave digest of the materials in nitric acid (67-69%, trace metal analysis grade) to determine the palladium content. Photoelectron spectroscopy in air (PESA) measurements were recorded using a Riken Keiki PESA spectrometer (Model AC-2). Samples for PESA were prepared on ITO glass substrates. Steady state photoluminescence of the polymer/*E. coli* biohybrids was measured by a Shimadzu RF-5301PC fluorescence spectrometer at room temperature. Sample solutions were transferred into quartz cuvettes and degassed with nitrogen for all the photophysical measurements.

Results and Discussion

Gel Permeation Chromatography

Table S1. GPC data for all chloroform-soluble polymer fractions.

Polymer	M_n[a]/ g mol^{-1}	M_w[a] / g mol^{-1}	*Đ*[b]
LP1	13,900	24,700	1.8
LP2	25,500	48,900	1.9
LP3	125,900	219,800	1.7
LP4	33,500	87,900	2.6

[a] Obtained from gel permeation chromatography in THF calibrated against polystyrene standards; M_n: the number-weighted molecular weight, M_w: the mass-weighted molecular weight. [b] Dispersity, $Đ = M_w/M_n$.

Photophysical Properties and Residual Pd Content

Table S2. Photophysical properties of nanoparticle solution of polymer LP10, LP11, LP21, LP31, and LP41; residual Pd content of five polymers.

Polymer	λ_{onset}[a]/ nm	Optical gap[b] / eV	Pd[c] / ppm
LP10	417	2.97	1598
LP11	417	2.97	1694
LP21	488	2.55	4412
LP31	518	2.39	93
LP41	441	2.81	269

[a] Absorption onset wavelength of polymer nanoparticles. [b] Optical gap calculated from absorption onset of polymer nanoparticles. [c] Obtained from inductively coupled plasma optical emission spectrometry.

Powder X-Ray Diffraction

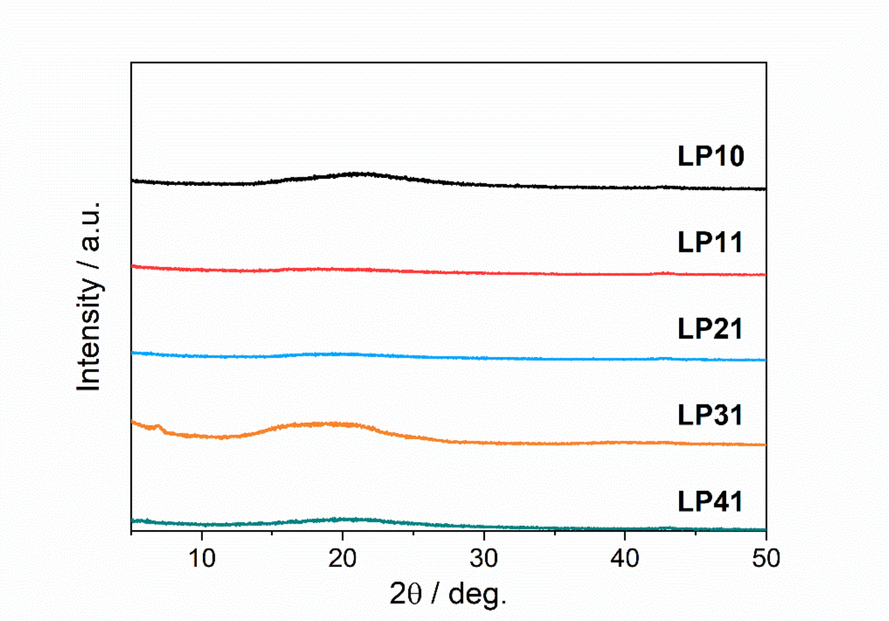

Figure S1. PXRD patterns of powders of polymer LP10, LP11, LP21, LP31, and LP41.

Thermogravimetric Analysis

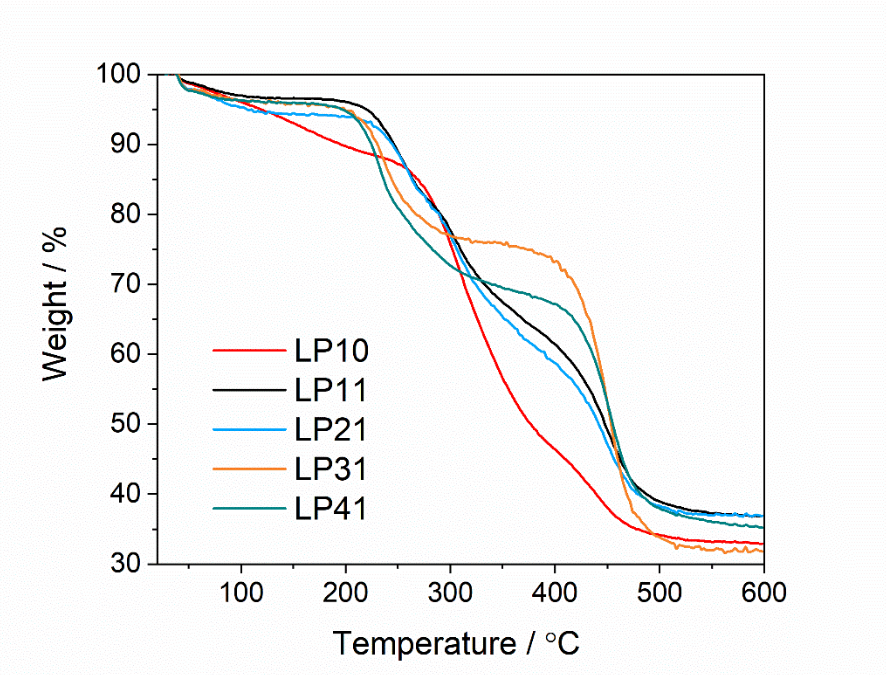

Figure S2. Thermogravimetric analysis of LP10, LP11, LP21, LP31, and LP41 under nitrogen at a heating rate of 10 °C min^{-1}.

UV-Vis and Photoluminescence Spectra

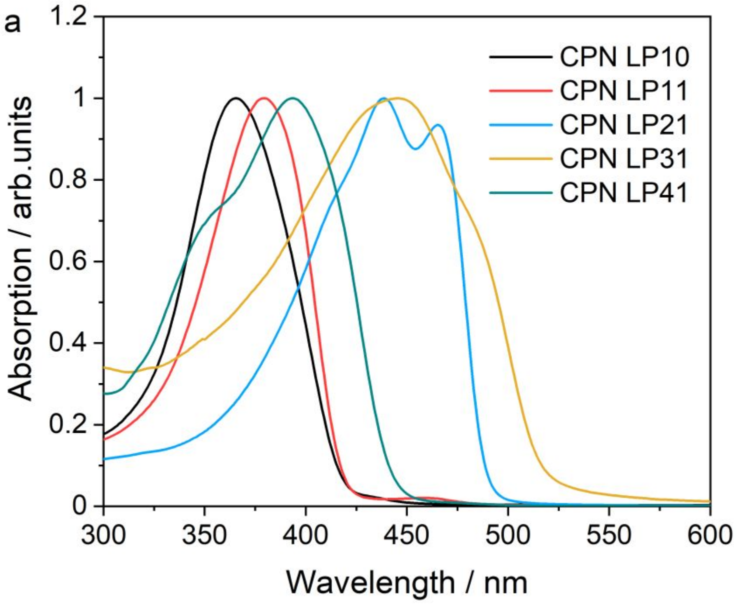

Figure S3a. Normalised UV-Vis absorption spectra of polymer nanoparticle aqueous solution.

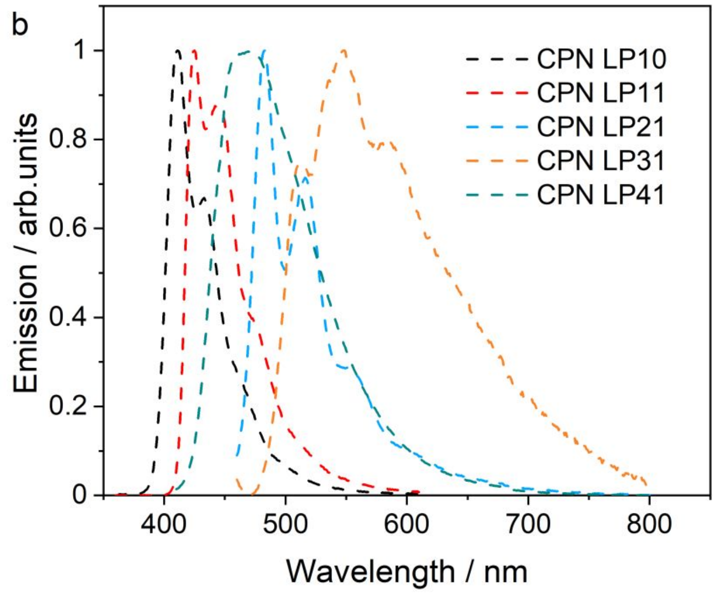

Figure S3b. Normalised photoluminescence emission spectra of polymer nanoparticle aqueous solution. λ_{exc} = 370 nm CPN LP10 and 11, λ_{exc} = 400 nm for the rest polymer nanoparticles.

Size Distribution and Zeta-potential

Table S3. Particle average size and zeta-potential (polymer conc. 50 mg L^{-1}) by dynamic light scattering.

Polymer	Average size/ nm	PDI[a] / eV	Zeta-potential / mV
LP10	161	0.293	41.4 ± 5.3
LP11	151	0.334	34.5 ± 5.8
LP21	258	0.404	39.1 ± 5.6
LP31	206	0.148	28.8 ± 4.0
LP41	95	0.364	56.1 ± 5.0

[a] PDI: polydispersity index.

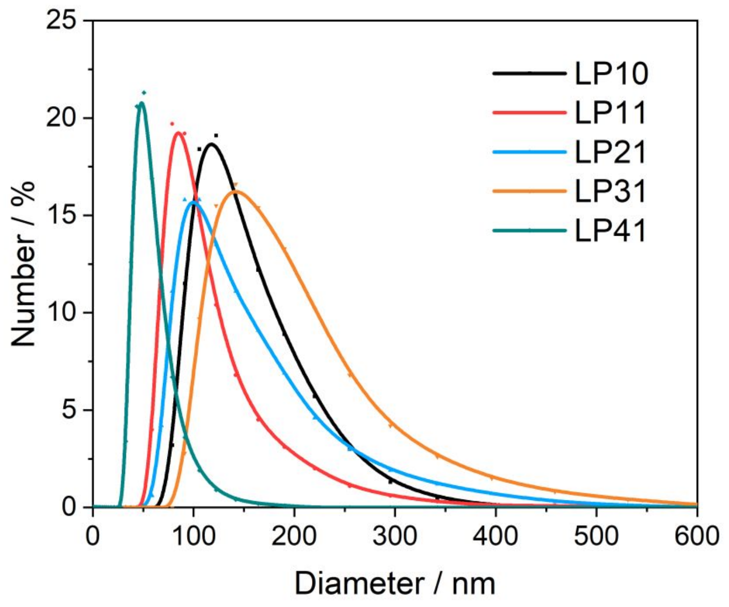

Figure S4. Size distribution of nanoparticle solution of 50 mg L^{-1} polymer LP10, LP11, LP21, LP31, and LP41 by number.

Scanning Electron Microscopy

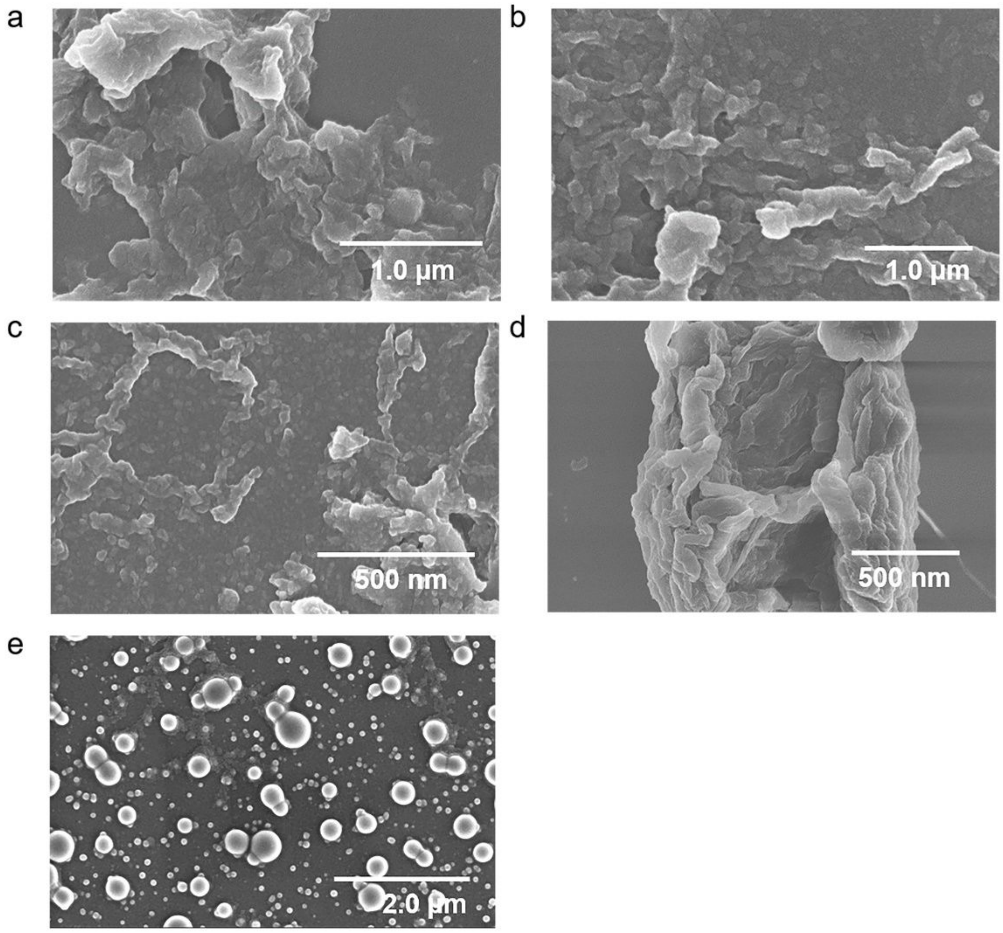

Figure S5. SEM images of (a) LP10, (b) LP11, (c) LP21, (d) LP31, and (e) LP41 polymer nanoparticles.

Figure S6. SEM images of HydA *E. coli* (200 µL in 10 mM Tris-HCl, final OD600 = 1.0) incubated with (a) LP10, (b) LP11, (c) LP21, and (d) LP31 nanoparticles (2.0 mL, 10 mg L^{-1}).

Figure S7. SEM images of LP41/*E. coli* after 3-h irradiation with a solar simulator.

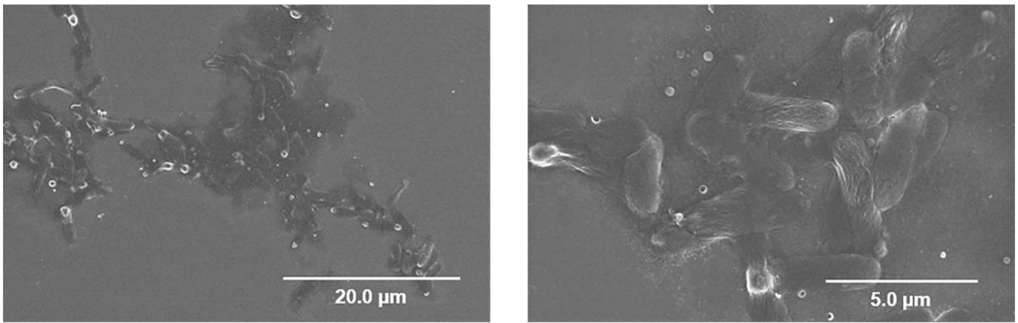

Confocal Fluorescence Microscopy

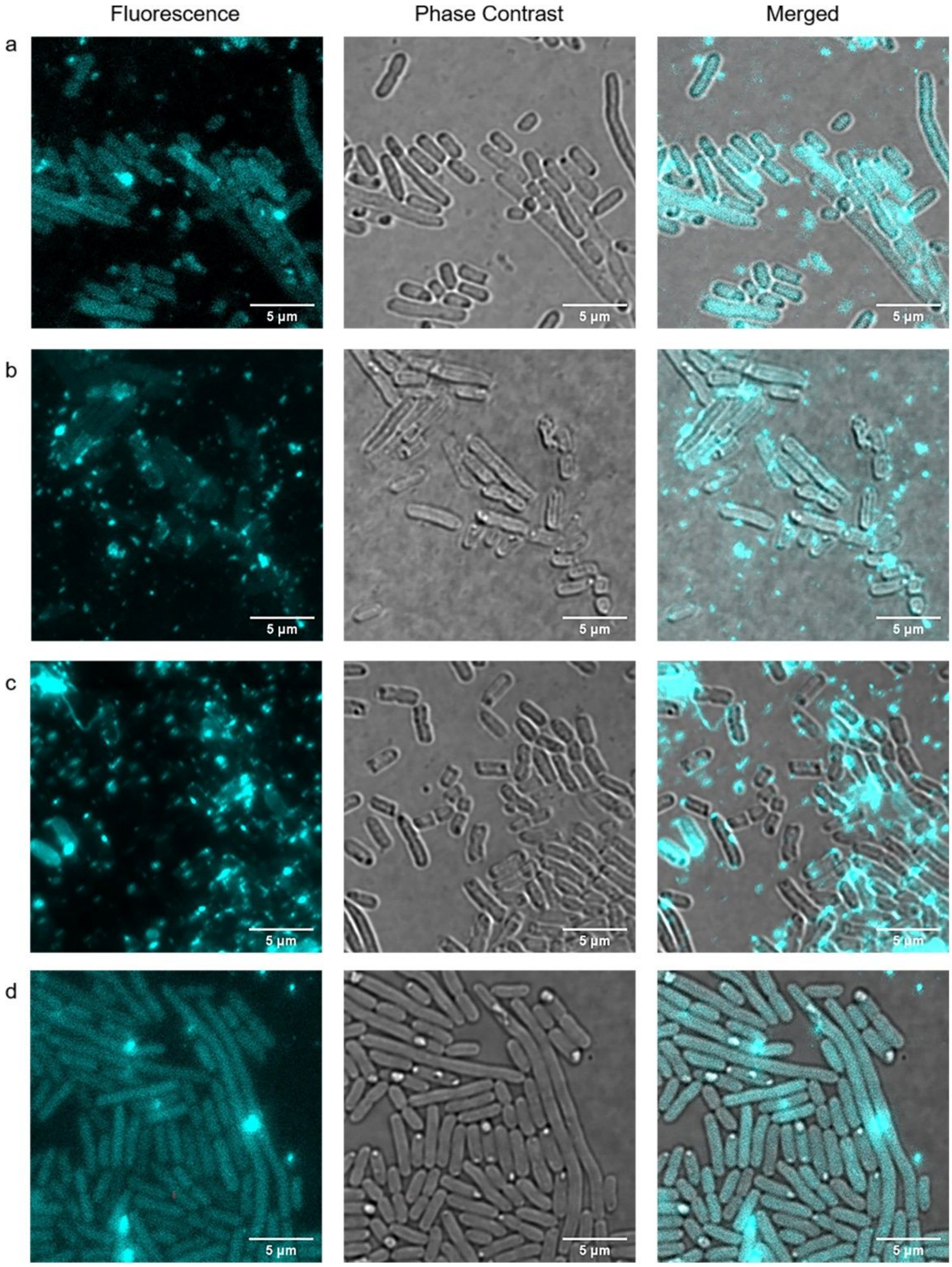

Figure S8. Confocal images of HydA *E. coli* (100 μL in 10 mM Tris-HCl, final OD600 = ~1.0) incubated with (a) LP10, (b) LP11, (c) LP21, and (d) LP31 nanoparticles (1.0 mL, 5 mg L^{-1}) for 5 mins (λ_{exc} = 488 nm).

Photoelectron Spectroscopy in Air (PESA)

Table S4. Photoelectron spectroscopy in air data for polymer thin-films.

Polymer	UV intensity / nW	Work function[a] / eV	PESA-infered ionization potentials[b] vs. SHE[c] / V
LP1	20	5.77	+ 1.33
LP2	20	5.53	+ 1.09
LP3	20	5.51	+ 1.07
LP4	100	5.89	+ 1.45

[a] Work functions determined using a Riken Keiki PESA spectrometer (Model AC-2) with a power number of 0.33. Samples were prepared on ITO glass substrates. [b] Obtained using the formula: IP = (work function - 4.44 eV). [c] SHE: Standard hydrogen electrode.

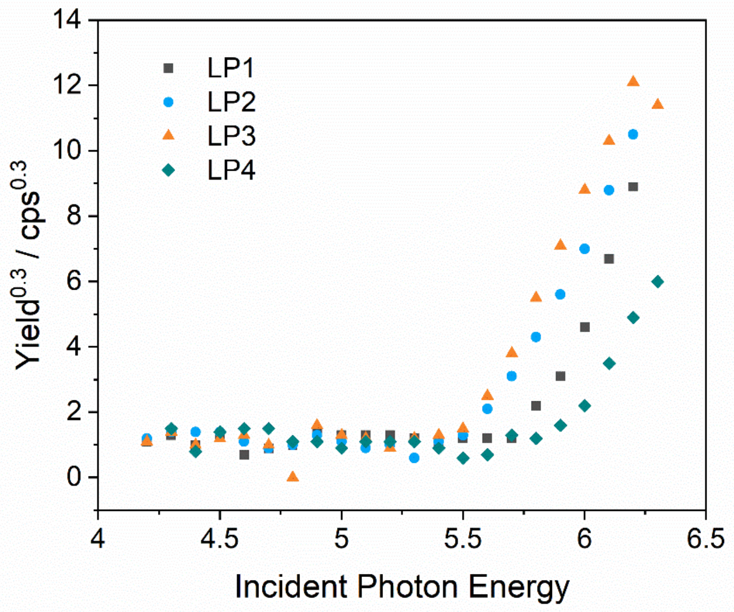

Figure S9. PESA spectra of LP1. LP2, LP3, and LP4 polymers.

(TD-)DFT Calculations

Table S5. Predicted charge carrier (IP, EA) and excitons (IP*, EA*) potentials of the polymers considered predicted using (TD-)B3LYP for oligomer models in water (ε_r 80.1) and under PESA conditions (ε_r 2.0). Data for LP1 and LP4 data in water was taken from previous work.[1]

Polymer	IP vs. SHE / V (ε_r 80.1)	EA vs. SHE / V (ε_r 80.1)	IP vs. SHE / V (ε_r 2.0)	EA* vs. SHE / V (ε_r 80.1)	IP* vs. SHE / V (ε_r 80.1)
LP1	0.73	-2.38	1.19	n/a[a]	n/a
LP2	0.46	-2.14	1.00	n/a	n/a
LP3	0.44	-2.11	0.89	n/a	n/a
LP4	1.00	-1.92	1.55	0.92	-1.73

[a] n/a: not applicable

Table S6. Predicted potentials for the different solution half-reactions at pH 0 were taken from previous work,[2] potentials at pH 6.5 were estimated based on Nernst equation[3]: $E = E^0 - 0.05916 \times pH$, E^0: potential at pH 0.

Solution half-reaction	Potential (V vs. SHE)	
	pH 0	pH 6.5
H+ (aq) + e- -> 1/2 H_2 (g)	0	-0.38
Ascorbic acid*[a] (aq) + H^+ (aq) + e^- -> Ascorbic acid (aq)	0.80	0.42
Dehydroascorbic acid[b] + 2 H^+ (aq) + 2e^- -> Ascorbic acid (aq)	0.40	0.015

[a] Ascorbic acid*: one-hole products of ascorbic acid. [b] Dehydroascorbic acid: two-hole oxidation products of ascorbic acid.

Photobiocatalytic Hydrogen Production

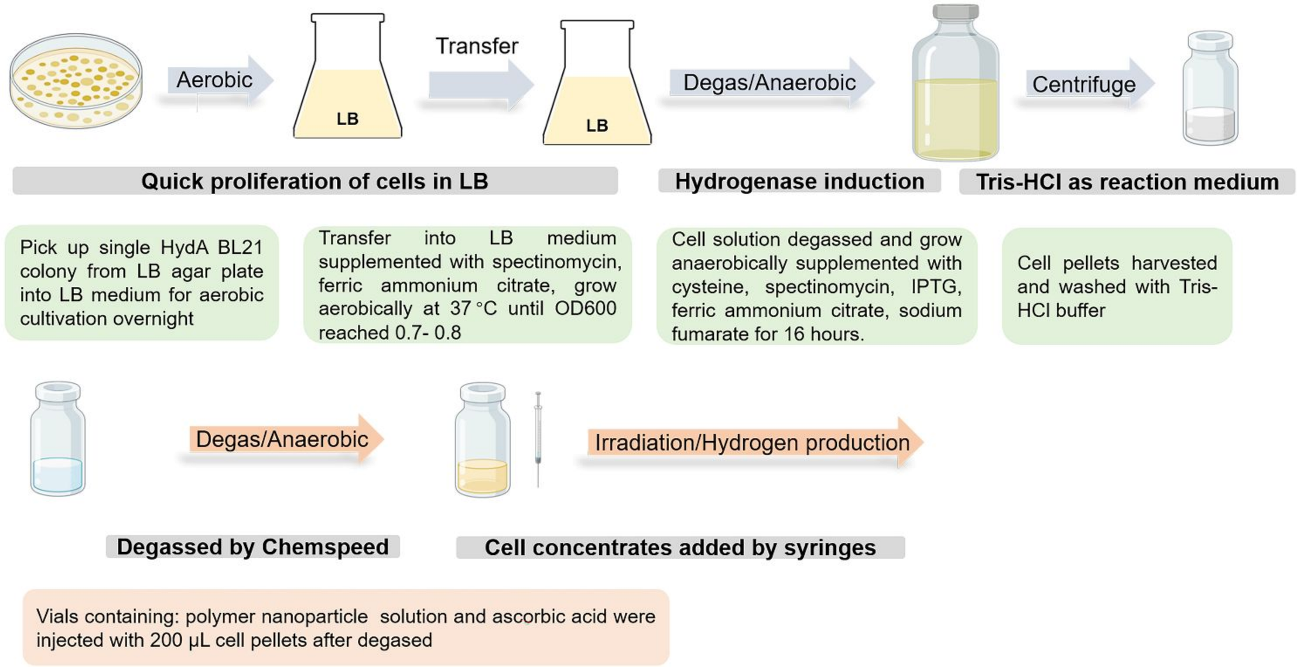

Figure S10. Strategy of hydrogenase induction, biohybrid system assembly, and hydrogen production. Images were created by BioRender.com.

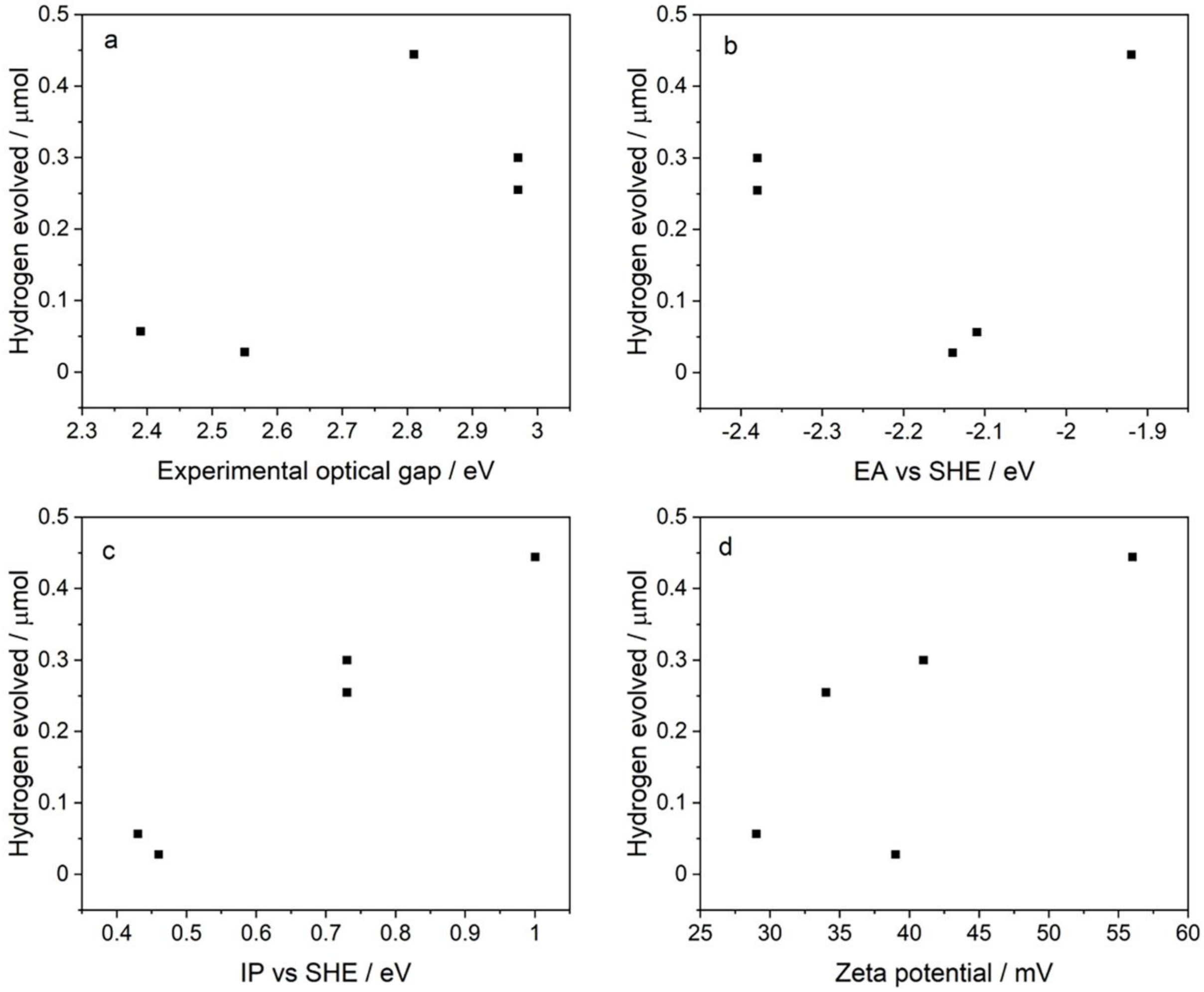

Figure S11. Correlation between the biohybrids' hydrogen evolution activity and the polymer optical gap (a) / EA value (b) / IP value (c)/zeta-potential (d).

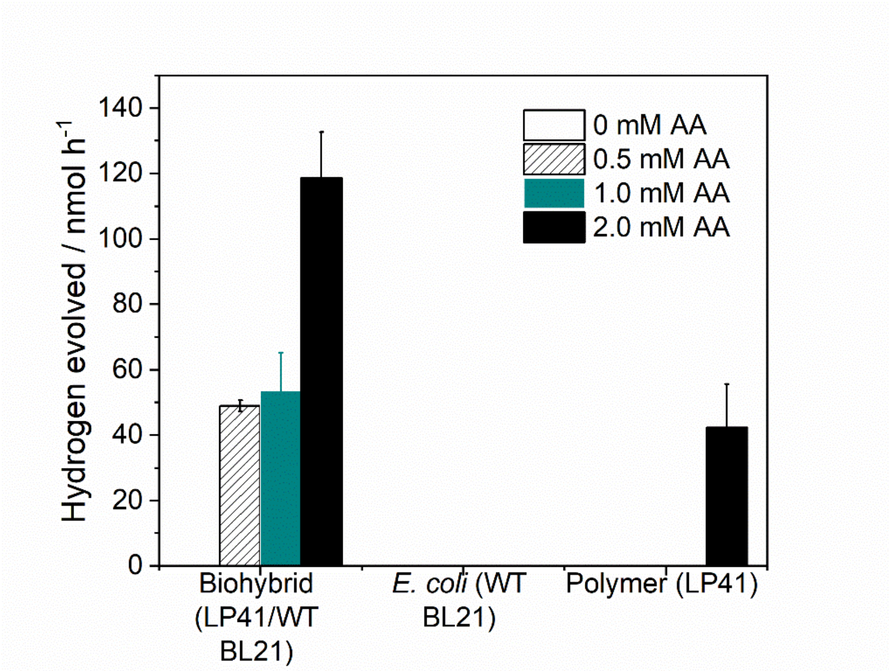

Figure S12. Hydrogen production performance of LP41/WT BL21 biohybrid systems with 0, 0.5, 1.0, and 2.0 mM ascorbic acid concentrations. All of the hydrogen production reactions were conducted in 10 mM tris(hydroxymethyl)aminomethane chloride buffer (pH 7, Tris-HCl) under irradiation of an AM 1.5G solar simulator for 3 h. Biohybrid reactions consisted of 4.3 mL polymer nanoparticle solution, 200 µL *E. coli* (final OD600 = ~ 1.0), 0.5 mL 100 mM Tris-HCl, and 50 µL ascorbic acid. Plots and error bars represent the averages and standard deviations of at least two assays.

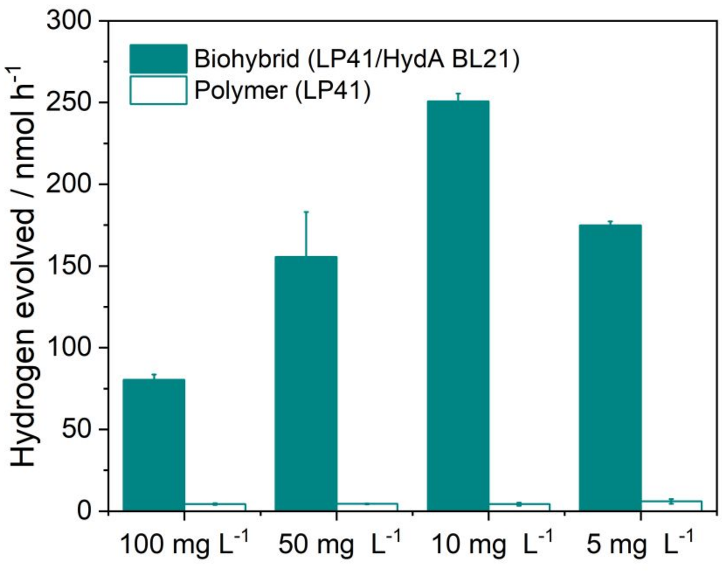

Figure S13. Hydrogen production performance of LP41/HydA BL21 biohybrid systems with 5, 10, 50, and 100 mg L^{-1} polymer concentrations. All of the hydrogen production reactions were conducted in 10 mM tris(hydroxymethyl)aminomethane chloride buffer (pH 7, Tris-HCl) under irradiation of an AM 1.5G solar simulator for 3 h. Biohybrid reactions consisted of 4.3 mL polymer nanoparticle solution, 200 µL *E. coli* (final OD600 = ~ 1.0), 0.5 mL 100 mM Tris-HCl, and 50 µL ascorbic acid. Plots and error bars represent the averages and standard deviations of at least two assays.

Photoluminescence Spectra

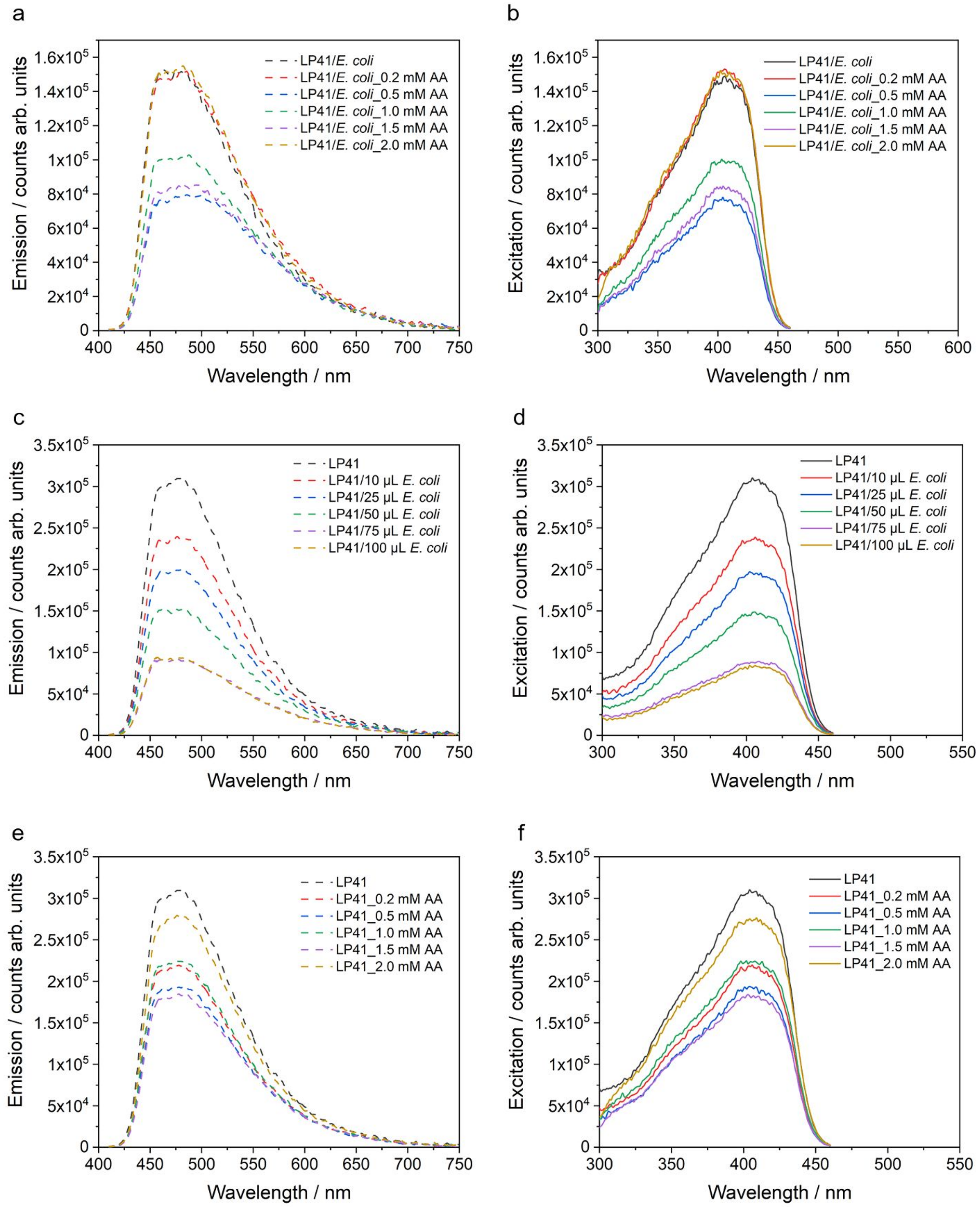

Figure S14. Emission (a) and excitation (b) spectrum of LP41/*E. coli* biohybrid systems with different ascorbic acid concentrations; Emission (c) and excitation (d) spectrum of LP41/*E. coli* biohybrid systems with different *E. coli* amounts; Emission (e) and excitation (f) spectrum of LP41 sample with different ascorbic acid concentrations. Excitation wavelength is 400 nm, and emission wavelength was 470 nm for all measurements.

Stern-Volmer Analysis

Stern-Volmer equation[4]:

$$\frac{I_0}{I} = 1 + \mathrm{k_q}\tau_0|Q| = 1 + \mathrm{K_D}|Q|$$

I_0: the fluorescence intensity in the absence of quencher

I: the fluorescence intensity in the presence of quencher

k_q: the biomolecular quenching constant

τ_0: the lifetime of the fluorophore in the absence of quencher

Q: the concentration of quencher

K_D: Stern-Volmer quenching constant, $K_D = k_q\tau_0$

Table S7 Stern-Volmer analysis of three sets of samples: LP41 with different amounts of *E. coli*, LP41/*E. coli* with different AA concentrations, and LP41 with different AA concentrations. I_0 / I: the inverse of normalized emission intensity at 470 nm; $C_{quencher}/C_{quencher,0}$: the relative equivalence of quenchers.

Sample	I_0/I	$C_{quencher}/C_{quencher,0}$
LP41	1.000	0
LP41/10 µL *E. coli*	1.273	0.10
LP41/25 µL *E. coli*	1.529	0.25
LP41/50 µL *E. coli*	2.058	0.50
LP41/75 µL *E. coli*	3.365	0.75
LP41/100 µL *E. coli*	3.225	1.0
LP41/ 50 µL *E. coli*	1.000	0
LP41/50 µL *E. coli*_0.2 mM AA	1.007	0.10
LP41/50 µL *E. coli*_0.5 mM AA	1.922	0.25
LP41/50 µL *E. coli*_1.0 mM AA	1.451	0.50
LP41/50 µL *E. coli*_1.5 mM AA	1.753	0.75
LP41/50 µL *E. coli*_2.0 mM AA	0.947	1.0
LP41	1.000	0
LP41_0.2 mM AA	1.392	0.1
LP41_0.5 mM AA	1.580	0.25
LP41_1.0 mM AA	1.373	0.50
LP41_1.5 mM AA	1.678	0.75
LP41_2.0 mM AA	1.107	1.0

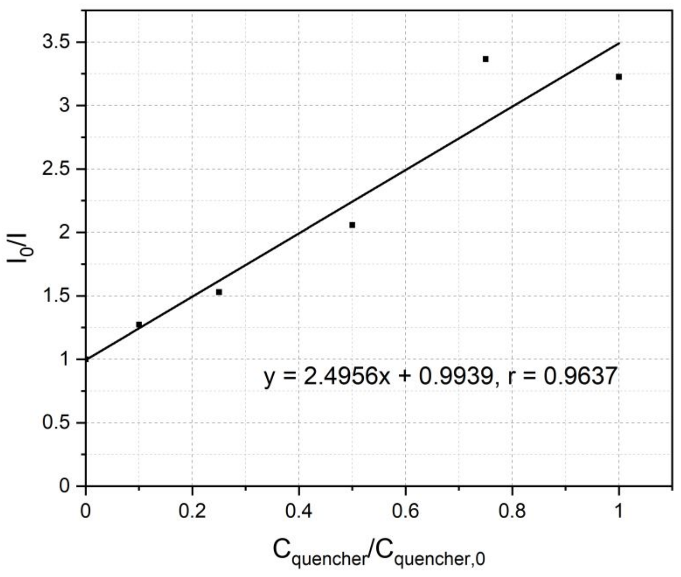

Figure S15 Linear relationship between I_0/I and $C_{quencher}/C_{quencher,0}$ of LP41/*E. coli* biohybrid systems with different amounts of *E. coli* concentrates. $C_{quencher}/C_{quencher,0}$: the relative equivalence of quenchers.

Time Correlated Single Photon Counting

Table S8. Estimated fluorescence lifetimes for biohybrid systems with different *E. coli* amounts (1-6); and estimated fluorescence lifetimes with different ascorbic acid concentrations (7-12, with 50 µL *E. coli*); and estimated fluorescence lifetimes for LP41 nanoparticle solution with different ascorbic acid concentrations (13-18). All samples are in 10 mM Tris-HCl buffer with 10 mg/L LP41 polymer concentration.

		τ_1/ ns	SD_1[a] / ns	B_1 / %	τ_2 / ns	SD_2 / ns	B_2 / %	τ_3 / ns	SD_3 / ns	B_3 / %	χ^2	τ_{AVG}[b] / ns	SD[c] / ns
1	LP41	0.66	0.02	59.32	2.20	0.07	32.69	11.58	0.50	7.99	1.17	2.04	0.07
2	LP41/10 µL *E. coli*	0.56	0.02	57.73	1.85	0.06	34.05	10.04	0.36	8.23	1.24	1.78	0.06
3	LP41/25 µL *E. coli*	0.50	0.02	61.10	1.85	0.05	32.30	10.49	0.46	6.60	1.18	1.60	0.06
4	LP41/50 µL *E. coli*	0.45	0.01	69.09	1.66	0.06	25.73	9.11	0.48	5.18	1.14	1.21	0.05
5	LP41/75 µL *E. coli*	0.49	0.01	80.62	1.50	0.09	15.99	7.54	0.48	3.39	1.26	0.89	0.04
6	LP41/100 µL *E. coli*	0.40	0.01	74.51	1.46	0.06	21.92	8.42	0.53	3.58	1.30	0.92	0.04
7	LP41/50 µL *E. coli*	0.45	0.01	69.09	1.66	0.06	25.73	9.11	0.48	5.18	1.14	1.21	0.05
8	LP41/*E. coli*_0.2 mM AA	0.47	0.01	69.28	1.72	0.06	26.36	9.06	0.54	4.35	1.21	1.18	0.05
9	LP41/*E. coli*_0.5 mM AA	0.33	0.01	67.45	1.18	0.04	27.75	6.68	0.32	4.80	1.26	0.87	0.03
10	LP41/*E. coli*_1.0 mM AA	0.42	0.01	73.38	1.46	0.06	23.41	7.72	0.55	3.21	1.21	0.90	0.04
11	LP41/*E. coli*_1.5 mM AA	0.44	0.01	77.73	1.29	0.09	18.80	5.34	0.39	3.46	1.36	0.77	0.04
12	LP41/*E. coli*_2.0 mM AA	0.49	0.02	73.40	1.46	0.08	23.29	6.37	0.47	3.31	1.22	0.91	0.05
13	LP41	0.66	0.02	59.32	2.20	0.07	32.69	11.58	0.50	7.99	1.17	2.04	0.07
14	LP41_0.2 mM AA	0.69	0.01	76.55	2.39	0.10	19.63	11.45	0.75	3.83	1.24	1.44	0.06
15	LP41_0.5 mM AA	0.48	0.01	64.31	1.64	0.05	31.19	8.08	0.42	4.67	1.58	1.20	0.04
16	LP41_1.0 mM AA	0.34	0.02	53.05	1.17	0.04	41.18	4.73	0.25	5.77	1.26	0.94	0.04
17	LP41_1.5 mM AA	0.33	0.02	56.43	1.07	0.04	37.86	4.26	0.22	5.71	1.22	0.84	0.04
18	LP41_2.0 mM AA	0.38	0.02	54.33	1.16	0.04	40.57	5.47	0.25	5.09	1.29	0.96	0.04

[a] SD: Standard deviation for τ. [b] Fluorescence life-times for all samples obtained from fitting time-correlated single photon counting decays to a sum of three exponentials, which yield τ_1, τ_2, and τ_3 according to $\sum_{i=1}^{n}\left(A + B_i exp\left({}^{-t}/_{\tau_i}\right)\right)$. τ_{AVG} is the weighted average lifetime calculated as $\sum_{i=1}^{n} B_i\tau_i$. [c] SD for τ_{AVG} is calculated as $\sum_{i=1}^{n} B_i SD_i$

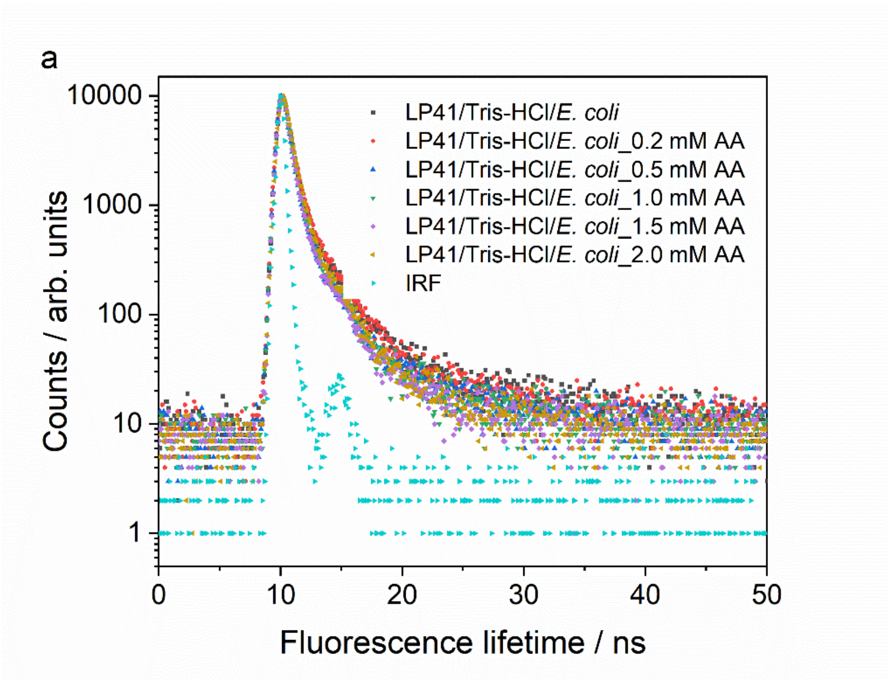

Figure S16a. Decay curves of LP41/*E.coli* biohybrid systems with different ascorbic acid concentrations with instrument response function (IRF).

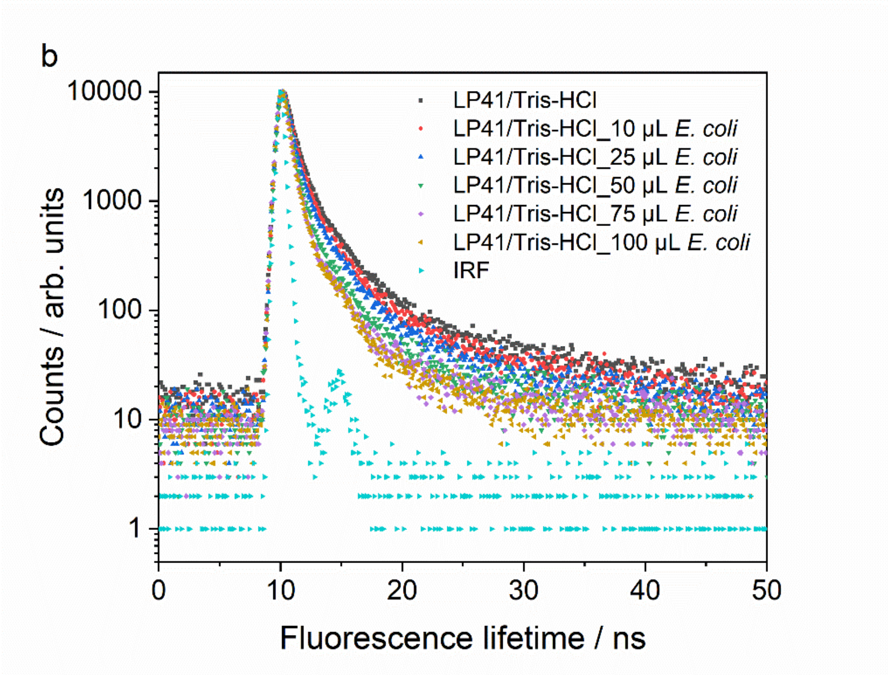

Figure S16b. Decay curves of LP41/*E.coli* biohybrid systems with different *E. coli* amounts with instrument response function (IRF).

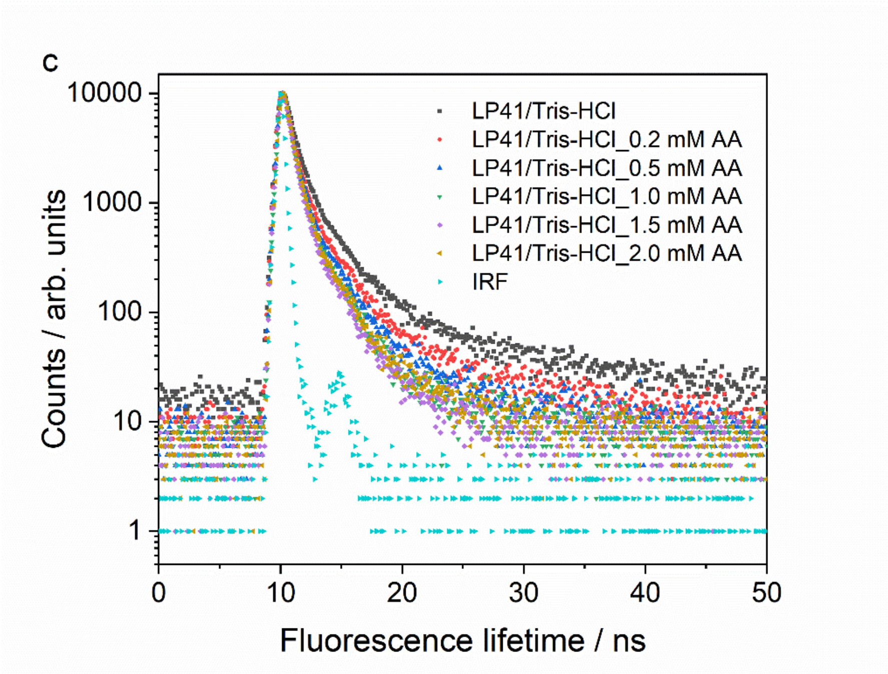

Figure S16c. Decay curves of LP41 with different ascorbic acid concentrations with instrument response function (IRF).

Transient Absorption Measurements

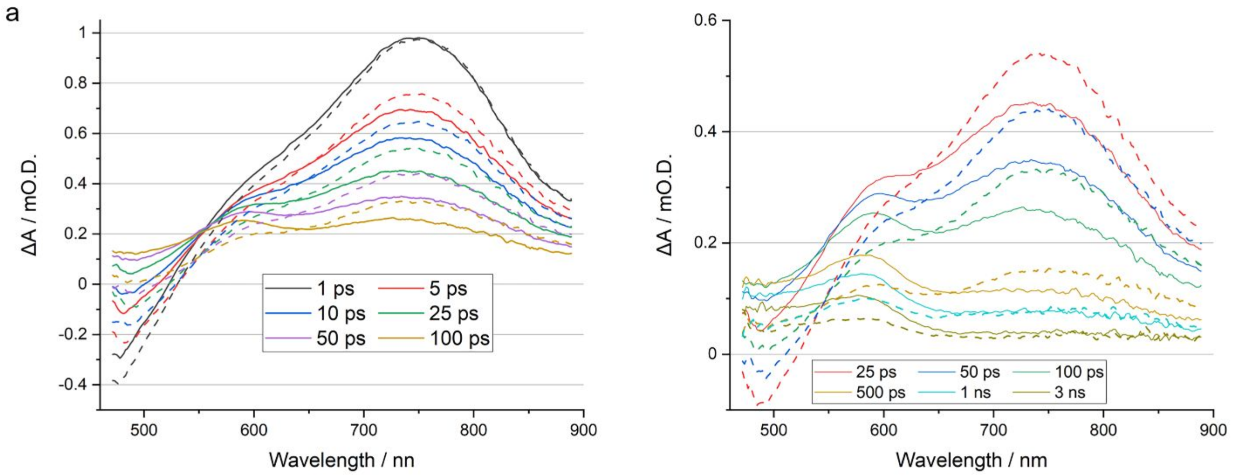

Figure S17a. TA spectra normalized at the global maximum (ΔA) for 10 mg/L LP41 polymer nanoparticle in Tris-HCl aqueous solution (solid line) and 10 mg/L LP41 polymer nanoparticle in Tris-HCl aqueous solution with 1 mM ascorbic acid (dashed line) at key pump-probe delays as indicated.

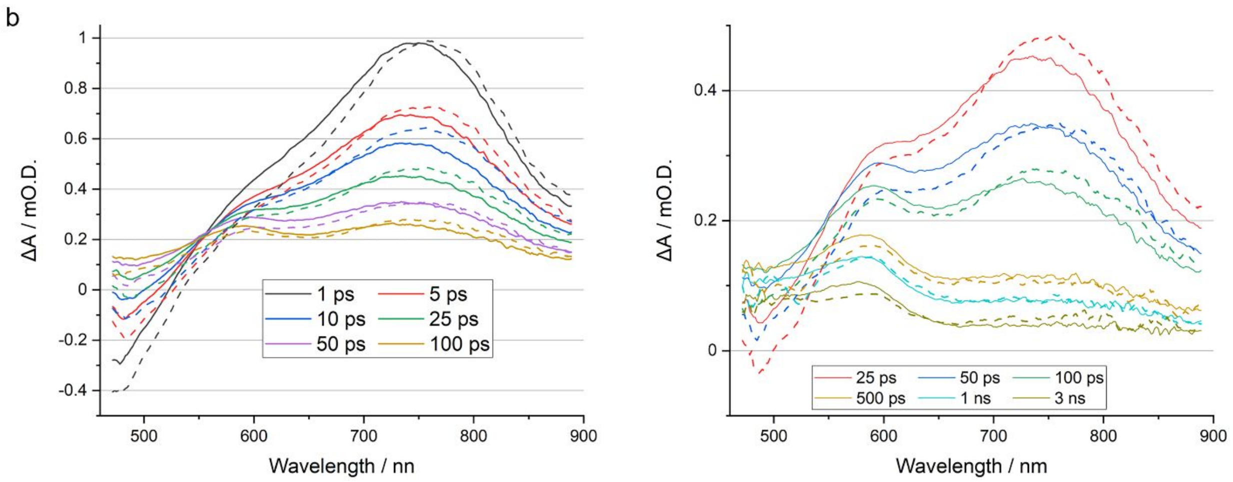

Figure S17b. TA spectra normalized at the global maximum (ΔA) for 10 mg/L LP41 polymer nanoparticle in Tris-HCl aqueous solution (solid line) and 10 mg/L LP41 polymer nanoparticle/25 µL *E. coli* in Tris-HCl aqueous solution (dashed line) at key pump-probe delays as indicated.

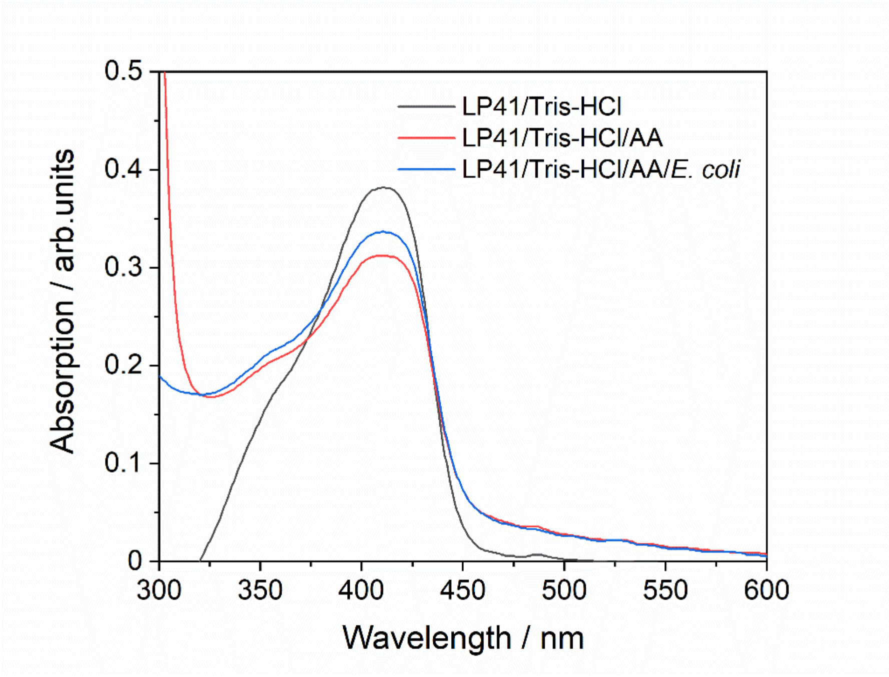

Figure S18. UV-vis spectra of samples conducted for TA measurements, 10 mg/L LP41 polymer nanoparticle in Tris-HCl aqueous solution (with/without ascorbic acid, with/without 25 µL *E. coli*).

Previous Literature Reports on Material-Microorganism Biohybrids for Hydrogen Production

Table S9. Reported material-microorganism biohybrid systems for hydrogen production.

Material	Microorganism	Reaction solution	Energy source	Light Source	Activity	Ref.
Bacterially precipitated CdS	*S. oneidensis MR-1*[a]	50 mM HEPES[b], 50 mM NaCl, 20 mM sodium lactate	Sodium lactate, light	300 W Xe lamp with a 420 nm cut-off filter	362.44 ± 119.69 μmol mg^{-1} hydrogen produced over 72 hours	5
Cu_2O/Reduced graphene oxide	*S. oneidensis MR-1*	100 mM Tris-HCl, 150 mM NaCl, 5% (v/v) glycerol and 100 mM ascorbic acid	Glycerol, light	300 W Xe lamp with a 420 nm cut-off filter	322.0 μmol/g_{Cu2O} within 4 h	6
Water-soluble dyes, inorganic complexes	*S. oneidensis*	50 mM HEPES, 50 mM NaCl, pH 7	Light	KL5125 cold light	725 ± 97 nmol of H_2 over 30 min with sodium dithionite and MV[c]	7
Bacterially precipitated CdS	*E. coli* K-12	SBC medium with 1 mM cysteine, 20 mM glucose	Glucose, light	300 W Xe lamp with bandpass filters	> 1.8 mmol H_2 over 3 hours	8
Bacterially precipitated CdS	Engineered *E. coli* BL21	100 mM Tris-HCl (pH 7), 150 mM NaCl, 5% glycerol, 100 mM ascorbic acid, 5 mM MV	Glycerol, light	350 W Xe lamp	13.4 μmol at 6 h, 81.8 μmol at 24 h by 10^8 cells	9
Bacterially precipitated CdS	Engineered *E. coli* BL21	100 mM BisTris-HCl (pH 5), 5 mM MV	Light	Monochromatic LED light	AQY[d]: 0.11, 0.10, and 0.04% at 385, 420, and 470 nm	10
Bacterially Precipitated $AgInS_2/In_2S_3$	*E. coli* MG1655	SBC medium with 1 mM cysteine, 20 mM glucose	Glucose, light	300 W Xe lamp	2.2 mmol-H_2/mmol-glucose at 2000 W m^{-2}	11
Biogenic $CdSe_xS_{1-x}$ nanoparticles	*E. coli*	3 mL LB[e] medium (pH 7, with 100 mM Tris-HCl buffer), 8 g/L glucose	LB, glucose, light	300 W Xe lamp with a 420 nm cut-off filter	2.9- fold higher hydrogen production rate than the pure *E. coli*	12
Anatase TiO_2 powder	Engineered *E. coli*	100 mM Tris-HCl (pH 7), 150 mM NaCl, 5 mM methyl viologen, 5% glycerol, 100 mM ascorbic acid	Glycerol, light	Xe lamp	0.72 μmol min^{-1} (mg wet cell)$^{-1}$ H_2	13
TiO_2 (P-25)	Engineered *E. coli* BL21	100 mM Tris-HCl (pH 7), 5 mM methyl viologen	Light	300 W Xe lamp with bandpass filters	AQY_{300}[f]: 26.4%, AQY_{350}: 31.2%	14
TiO_2	*E. coli* K-12	100 mM Tris-HCl (pH 7), 150 mM NaCl, 5 mM methyl viologen, 6 mM glucose, 100 mM ascorbic acid	Glucose, light	Xe lamp	A 2-fold increase in the hydrogen production compared to that without TiO_2	15
Iodine-doped hydrothermally carbonized carbon	*E. coli*-K12 (MG1655)	Simplified BC medium, 20 mm glucose, 1 mM cysteine	Glucose, light	Xe lamp	Quantum efficiency of 9.11%	16

[a] *S. oneidensis*: *Shewanella oneidensis*. [b] HEPES: 4-(2-hydroxyethyl)-1-piperazineethanesulfonicacid. [c] MV: Methyl viologen. [d] AQY: apparent quantum yield. [e] LB: Lysogeny broth. [f] AQY_{300}: apparent quantum yield at 300

External Quantum Efficiency (EQE) Measurements

Table S10. External quantum efficiencies of LP41 / HydA *E. coli* biohybrid system (6.8 mL 10 mg L^{-1} LP41 with 300 µL HydA *E. coli* pellet with 1 mM ascorbic acid in 10 mM Tris-HCl) at specified wavelengths.

λ / nm	LED power [a] / mW	Total evolved H_2[b] / µmol	EQE[c] / %
**395**	7.6	0.7	0.08
**420**	16.8	1.0	0.05

[a] Determined using a Thor Labs sensor (sensor area = 0.709 cm^2). [b] Over a 90-minute period of illumination by monochromatic LED. [c] Determined using the equation below.

$$EQE\ (\%) = \frac{2 \times n_{H_2}}{n_{photons}} (\times 100\%) = \frac{2 \times n_{H_2} \times \mathrm{N_A} \times \mathrm{h} \times \mathrm{c} \times A_S}{t \times \lambda \times P_S \times A_W} (\times 100\%)$$

Where n_{H2} is the number of moles of hydrogen, $n_{photons}$ is the number of moles of photons, N_A is Avogadro's constant, h Planks' constant, c is the speed of light in a vacuum, A_S is the area of the sensor, t is the illumination time, λ is the wavelength of the LED, P_S is the power observed at the sensor, and A_W is the area of the window of the quartz cuvette used (2 cm × 4 cm

References

(1) Bai, Y.; Woods, D. J.; Wilbraham, L.; Aitchison, C. M.; Zwijnenburg, M. A.; Sprick, R. S.; Cooper, A. I. Hydrogen Evolution from Water Using Heteroatom Substituted Fluorene Conjugated Co-Polymers. *J. Mater. Chem. A* **2020**, *8* (17), 8700–8705.

(2) Wang, X.; Chen, L.; Chong, S. Y.; Little, M. A.; Wu, Y.; Zhu, W.-H.; Clowes, R.; Yan, Y.; Zwijnenburg, M. A.; Sprick, R. S.; Cooper, A. I. Sulfone-Containing Covalent Organic Frameworks for Photocatalytic Hydrogen Evolution from Water. *Nat. Chem.* **2018**, *10* (12), 1180–1189.

(3) Feiner, A.-S.; McEvoy, A. J. The Nernst Equation. *J. Chem. Educ.* **1994**, *71* (6), 493.

(4) Lakowicz, J. R. *Principles of Fluorescence Spectroscopy*; Springer, 2006.

(5) Han, H.-X.; Tian, L.-J.; Liu, D.-F.; Yu, H.-Q.; Sheng, G.-P.; Xiong, Y. Reversing Electron Transfer Chain for Light-Driven Hydrogen Production in Biotic–Abiotic Hybrid Systems. *J. Am. Chem. Soc.* **2022**.

(6) Shen, H.; Wang, Y. Z.; Liu, G.; Li, L.; Xia, R.; Luo, B.; Wang, J.; Suo, D.; Shi, W.; Yong, Y. C. A Whole-Cell Inorganic-Biohybrid System Integrated by Reduced Graphene Oxide for Boosting Solar Hydrogen Production. *ACS Catal.* **2020**, *10* (22), 13290–13295.

(7) Rowe, S. F.; Le Gall, G.; Ainsworth, E. V.; Davies, J. A.; Lockwood, C. W. J.; Shi, L.; Elliston, A.; Roberts, I. N.; Waldron, K. W.; Richardson, D. J.; Clarke, T. A.; Jeuken, L. J. C.; Reisner, E.; Butt, J. N. Light-Driven H2 Evolution and C=C or C=O Bond Hydrogenation by Shewanella Oneidensis: A Versatile Strategy for Photocatalysis by Nonphotosynthetic Microorganisms. *ACS Catal.* **2017**, *7* (11), 7558–7566.

(8) Wang, B.; Zeng, C.; Chu, K. H.; Wu, D.; Yip, H. Y.; Ye, L.; Wong, P. K. Enhanced Biological Hydrogen Production from Escherichia Coli with Surface Precipitated Cadmium Sulfide Nanoparticles. *Adv. Energy Mater.* **2017**, *7* (20), 1–10.

(9) Wei, W.; Sun, P.; Li, Z.; Song, K.; Su, W.; Wang, B.; Liu, Y.; Zhao, J. A Surface-Display Biohybrid Approach to Light-Driven Hydrogen Production in Air. *Sci. Adv.* **2018**, *4* (2), eaap9253.

(10) Honda, Y.; Shinohara, Y.; Watanabe, M.; Ishihara, T.; Fujii, H. Photo-Biohydrogen Production by Photosensitization with Biologically Precipitated Cadmium Sulfide in Hydrogen-Forming Recombinant Escherichia Coli. *ChemBioChem* **2020**, *21* (23), 3389–3397.

(11) Jiang, Z.; Wang, B.; Yu, J. C.; Wang, J.; An, T.; Zhao, H.; Li, H.; Yuan, S.; Wong, P. K. AglnS2/In2S3 Heterostructure Sensitization of Escherichia Coli for Sustainable Hydrogen Production. *Nano Energy*

2018, *46* (January), 234–240.

(12) Cui, S.; Tian, L. J.; Li, J.; Wang, X. M.; Liu, H. Q.; Fu, X. Z.; He, R. L.; Lam, P. K. S.; Huang, T. Y.; Li, W. W. Light-Assisted Fermentative Hydrogen Production in an Intimately-Coupled Inorganic-Bio Hybrid with Self-Assembled Nanoparticles. *Chem. Eng. J.* **2022**, *428* (May 2021).

(13) Honda, Y.; Hagiwara, H.; Ida, S.; Ishihara, T. Application to Photocatalytic H2Production of a Whole-Cell Reaction by Recombinant Escherichia Coli Cells Expressing [FeFe]-Hydrogenase and Maturases Genes. *Angew. Chemie - Int. Ed.* **2016**, *55* (28), 8045–8048.

(14) Honda, Y.; Watanabe, M.; Hagiwara, H.; Ida, S.; Ishihara, T. Inorganic/Whole-Cell Biohybrid Photocatalyst for Highly Efficient Hydrogen Production from Water. *Appl. Catal. B Environ.* **2017**, *210*, 400–406.

(15) Ramprakash, B.; Incharoensakdi, A. Light-Driven Biological Hydrogen Production by Escherichia Coli Mediated by TiO2 Nanoparticles. *Int. J. Hydrogen Energy* **2020**, *45* (11), 6254–6261.

(16) Xiao, K.; Tsang, T. H.; Sun, D.; Liang, J.; Zhao, H.; Jiang, Z.; Wang, B.; Yu, J. C.; Wong, P. K. Interfacing Iodine-Doped Hydrothermally Carbonized Carbon with Escherichia Coli through an “Add-on” Mode for Enhanced Light-Driven Hydrogen Production. *Adv. Energy Mater.* **2021**, *11* (21), 1–13.
